# Ribosome-binding antibiotics increase bacterial longevity and growth efficiency

**DOI:** 10.1073/pnas.2221507120

**Published:** 2023-09-26

**Authors:** Emily Wood, Hinrich Schulenburg, Philip Rosenstiel, Tobias Bergmiller, Dyan Ankrett, Ivana Gudelj, Robert Beardmore

**Affiliations:** ^a^Biosciences, College of Life and Environmental Sciences, University of Exeter, Exeter EX4 4QD, United Kingdom; ^b^Engineering and Physical Sciences Research Council Hub for Quantitative Modelling in Healthcare, University of Exeter, Exeter EX4 4QJ, United Kingdom; ^c^Evolutionary Ecology and Genetics, Zoologisches Institut, Christian-Albrechts-Universität zu Kiel, Am Botanischen Garten 1-9, Kiel 24118, Germany; ^d^Instituts für Klinische Molekularbiologie, Dekanat der Medizinischen Fakultät, Christian-Albrechts-Universität zu Kiel, Christian-Albrechts-Platz 4, Kiel D-24118, Germany

**Keywords:** antibiotic resistance, ribosome, microbial evolution, microbial longevity, ROS

## Abstract

Antibiotics, by definition, reduce bacterial growth rates in optimal culture conditions; however, the real-world environments bacteria inhabit see rapid growth punctuated by periods of low nutrient availability. How antibiotics mediate population decline during these periods is poorly understood. Bacteria cannot optimize for all environmental conditions because a growth-longevity tradeoff predicts faster growth results in faster population decline, and since bacteriostatic antibiotics slow growth, they should also mediate longevity. We quantify how antibiotics, their targets, and resistance mechanisms influence longevity using populations of *Escherichia coli* and, as the tradeoff predicts, populations are maintained for longer if they encounter ribosome-binding antibiotics doxycycline and erythromycin, a finding that is not observed using antibiotics with alternative cellular targets. This tradeoff also predicts resistance mechanisms that increase growth rates during antibiotic treatment could be detrimental during nutrient stresses, and indeed, we find resistance by ribosomal protection removes benefits to longevity provided by doxycycline. We therefore liken ribosomal protection to a “Trojan horse” because it provides protection from an antibiotic but, during nutrient stresses, it promotes the demise of the bacteria. Seeking mechanisms to support these observations, we show doxycycline promotes efficient metabolism and reduces the concentration of reactive oxygen species. Seeking generality, we sought another mechanism that affects longevity and we found the number of doxycycline targets, namely, the ribosomal RNA operons, mediates growth and longevity even without antibiotics. We conclude that slow growth, as observed during antibiotic treatment, can help bacteria overcome later periods of nutrient stress.

## Significance

It is often assumed that antibiotics are detrimental to bacterial lifestyles. While there is literature describing alternative perspectives on the function of antibiotics, this belief has likely perpetuated because of the limited way laboratory tests are designed for antibiotic function whereby short-term growth inhibition assays are the norm, by far. We take a different approach and show, using long-term assays, that some antibiotic classes can benefit bacteria in several ways that we summarize: Thus, ribosome-binding antibiotics benefit bacteria by alleviating stress and helping mitigate population decline during death phases, maintaining bacterial populations at higher densities for longer compared to untreated populations.

Human medicine needs antibiotics to act as warfare molecules, which they do. However, many antibiotics are synthesized by microbes (1, 2) whereby small molecules exit producer cells and diffuse through the extracellular environment where they are more likely to encounter nearby kin than foe in spatially structured microenvironments. Given this, could antibiotics be multifunctional and benefit kin while also harming foe? Indeed, antibiotic synthesis typically occurs during times of stress, not during rapid growth when the impairment of competitors would seem most important. Moreover, bactericidal antibiotics that hasten the onset of death antagonize with bacteriostatic antibiotics (3), opening the possibility that bacteriostatic antibiotics are, somehow, antagonistic with respect to bacterial death per se. And, although hard to quantify, due to the immense size of natural microbial communities (4) a significant proportion of antibiotic-microbe interactions take place environmentally, outside the context of human medicine, which can maintain resistance genes well away from human interference (5). We therefore need better understanding of the gamut of environmental and ecological mechanisms that maintain resistance and we propose that a hitherto unobserved benefit of antibiotics may play a role in this.

If impeding bacterial growth is not an antibiotic’s sole purpose, what other functions might it have? As ribosome-binding antibiotics can scavenge reactive oxygen species (ROS) (6, 7), we sought bacterial traits that might benefit from this during periods of nutrient stress; we show population longevity can derive benefits from just a single antibiotic exposure. As a result, when assessing the long-term fates of resistant and susceptible bacterial subpopulations, we must account not only for evolution that overcomes reductions in growth rate (8–10) but we should also assess how resistance and susceptibility mediate population longevity.

Throughout, “population longevity” means the time for which a microbial population is maintained at a positive density whereby at least one cell has the potential to spawn new growth, the term does not refer to the life-span of individual cells (11). Similarly, a “death phase” is a decline in population density. Since population growth equals birth minus death, cell division can take place during death phases and cell death can occur during growth phases. Different measurement proxies are used to measure population growth and death in vitro, and here, we apply several methodologies using *Escherichia coli* as a model to study how antibiotics mediate population longevity.

## Prior Stress Coping Strategies

Tradeoffs can be mediated by antibiotics, for example, there is a growth-lysis tradeoff due to *β*-lactam antibiotics (12). Bacteria also exhibit a “growth–death tradeoff” whereby slower growth results in slower population decline (13, 14) and bacteriostatic antibiotics could mediate this tradeoff, possibly even to the benefit of bacteria. This possibility is poorly understood, in part because death phases are not quantified during standardized antibiotic susceptibility tests (ASTs) that measure growth over short periods that can be as little as 4 h (15). ASTs are performed in media that facilitate rapid growth but, by contrast, real-world microbes experience multifactorial stresses in energy-limited environments (16) where temporal fluctuations in nutrient availability cause stress, including in the human gut (17) where competition for resources is fierce (18–20). Given this, bacteria have many mechanisms for coping with stress.

The laws of thermodynamics that govern energy consumption and entropy accumulation all but force stress and death upon microorganisms. The ability of cells to mitigate the molecular dynamical processes that lead to death and, instead, sustain cellular integrity in nutrient-poor conditions is vital for survival until such time nutrients become plentiful again. Before that moment arises, bacteria can form spores (21), persister cells can withstand high antibiotic dosages and prolonged stresses (22) and populations can survive for years due to “growth advantage in stationary phase” (GASP) mutants (23, 24). Thus, increased longevity is a highly advantageous trait but little is known about how antibiotics mediate this, so we study it. We find that while ribosome-binding antibiotics can improve population longevity, this benefit can be lost in antibiotic-resistant populations if proteins protect the ribosome from antibiotics. Thus, resistance, if shared among bacteria by plasmids, could even act like a “Trojan-horse”.

We analyze *E.coli* strains, including the Keio collection of single gene knockouts, to determine other antibiotic-related benefits, ranging from the exploitation of extracellular metabolites during starvation, antibiotic-induced changes in metabolism to protection against ROS damage by ribosome-binding antibiotics (6, 25). This reveals a benefit from antibiotics which arises because certain Keio strains that cannot grow without antibiotics, due to a lack of protection from ROS stresses, have their growth restored by antibiotic treatment.

Finally, we show that it is not necessary to target the ribosome with antibiotics in order to mediate population longevity, rather, this can be done via the ribosome directly. We demonstrate this using *E. colirrn* strains with different numbers of ribosomal RNA operons that exhibit differential population longevity whereby one number of operons (7) optimizes growth rate but a smaller number optimizes population longevity.

## Results

### Ribosome-Binding Antibiotics Confer a Benefit to Longevity.

We now study the growth and decline of *E. coli* (MG1655) over extended periods to establish whether bacteriostatic antibiotics influence population longevity. We use ribosome-targeting antibiotics from different classes, doxycycline and erythromycin, and 2 non-ribosome-targeting antibiotics, penicillin and rifampicin. Erythromycin promotes the correction of protein errors during amino acid starvation in bacteria (26) so we reasoned it could mediate longevity. It also slows aging in *Saccharomyces cerevisiae* where it is thought to reduce protein synthesis errors and the generation of free radicals (27). We also used doxycycline because it has a different primary binding site in the ribosome to erythromycin.

MG1655 was cultured in a treatment–growth–death (TGD) protocol (Methods A) with liquid M9 media supplemented with 0.2% glucose and 0.1% casamino acids (M9CAA) with antibiotics used at two concentrations (3 replicates per dose) that inhibit 20% and 50% relative to 24-h drug-free growth (*SI Appendix*, Fig. S1). Cultures were left for 28 d without the addition of further media, nutrients, or antibiotics. In contrast to cultivation methods based on chemostats or serial transfers, this forces populations to pass through the phases of lag, exponential growth, stationary phase, and death without the removal of cell debris or extracellular metabolites.

Antibiotics are only deployed at inoculation so their concentration must decrease through time. Different antibiotics degrade at different rates (28–31) and our assays show doxycycline in M9 media continues to inhibit MG1655 until day 28, after which it is no longer inhibitory (*SI Appendix*, Fig. S2). When *E. coli* (MG1655) was exposed to 0.2 (IC${}_{20}$) and 0.4mg/L (IC${}_{50}$) doxycycline concentrations for 28 d, doxycycline concentration reduced by approximately 46% and 60% respectively (*SI Appendix*, Fig. S3). Now, *β*-lactams degrade more rapidly (28, 30) but comparisons between antibiotics in terms of how each mediates longevity over 28d (Methods A) are well-defined, despite these differences, because we only expose each population to each antibiotic once in the TGD protocol.

**Fig. 1.**
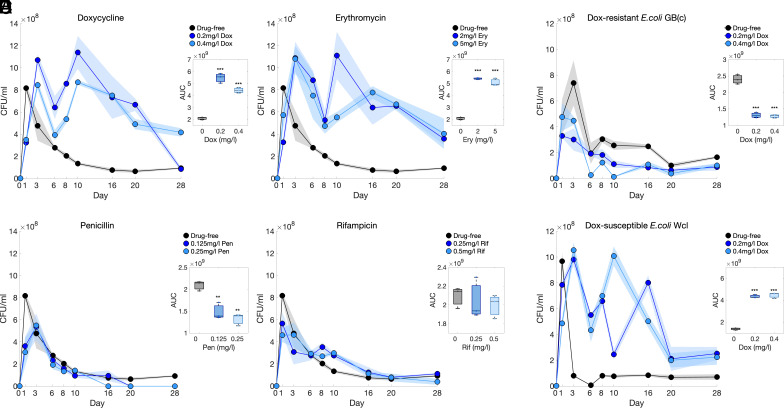
One treatment of a ribosome-binding antibiotic confers longevity benefits that are abolished by a resistance mechanism. The treatment–growth–death dynamics (TGD, Methods A) of MG1655 cultures over 28 d reveal longevity benefits from the ribosome-binding antibiotics (*A*) doxycycline (0.2 and 0.4 mg/L) and (*B*) erythromycin (2 and 5 mg/L). Those benefits are absent in the presence of non-ribosome-binding drugs (*C*) penicillin (0.125 and 0.25 mg/L) and (*D*) rifampicin (0.25 and 0.5 mg/L) and abolished by a doxycycline resistance mechanism (*E*; where *F* is a control for *E*). With no antibiotic exposure cell density decreases rapidly after day 1 (black lines in *A*, *B*, *C*, and *D*) but doxycycline (*A*) and erythromycin (*B*) exposure result in multiple phases of growth and decline with increased longevity. Areas under the curve (AUC, see all insets) are significantly increased for *A* and *B* (one-way ANOVA doxycycline: $\;{\text{F}}_{\left(2,6\right)}$ = 118.3, P< 0.0001, erythromycin: F${}_{\left(2,6\right)}$ = 251.5, P< 0.0001 with post hoc Tukey). In contrast, penicillin (*C*) and rifampicin (*D*) do not confer longevity benefits with a significant decrease in AUC using penicillin relative to drug-free (one-way ANOVA, F${}_{\left(2,6\right)}$ = 21.8, P<0.01, with post hoc Tukey) and no significant difference in AUC found between drug-free and rifampicin-treated populations. (*E*) Longevity benefits are absent in a doxycycline-resistant strain (*E. coli* (GB(c)) with a significant decrease of AUC in doxycycline-exposed populations (0.2 and 0.4 mg/L) relative to drug-free (one-way ANOVA: F${}_{\left(2,6\right)}$ = 119.07, P< 0.0001, with post hoc Tukey). (*F*) In contrast, a comparable doxycycline-sensitive strain (*E. coli* (Wcl)) does has longevity benefits with a significant increase in AUC due to doxycycline (0.2 and 0.4 mg/L) exposure (one-way ANOVA: F${}_{\left(2,6\right)}$ = 294.9, P< 0.001, with post hoc Tukey). Mean data are solid lines with ± estimated 95% CIs (shaded areas). Box plots (insets) show medians and first and third quartiles (asterisks indicate P values: ** = P<0.01, *** = P<0.001, *n* = 3).

As expected, population densities of MG1655 initially increase as nutrients are utilized (*SI Appendix*, Fig. S4). Densities then decrease monotonically after 24 h in drug-free conditions (Fig. 1, black lines) but the use of doxycycline (Fig. 1*A*) or erythromycin (Fig. 1*B*) results in a significant deviation from this: the monotone decease of density is replaced by oscillations whereby populations exhibit multiple phases of growth and decline (Fig. 1 *A* and *B*). Thus, doxycycline and erythromycin increase population longevity, quantified using an area under the curve (AUC) measure (Fig. 1 *A* and *B*). Longevity benefits are also observed at higher doxycycline concentrations, ones that inhibit approximately 90% of growth at 24 h (i.e., the IC${}_{90}$; *SI Appendix*, Fig. S5). Data suggest antibiotics stimulate efficient biomass production because the greatest realized population densities occur in drug-exposed conditions, despite nutrient availability being identical one-way ANOVA: doxycycline vs drug-free P<0.0001, erythromycin vs drug-free P<0.0001, Fig. 1 *A* and *B* (*Inset*).

Drug-induced longevity does not, however, extend to the 2 antibiotics with nonribosomal targets. Rather, the density dynamics of MG1655 exposed to penicillin (Fig. 1*C*) and rifampicin (Fig. 1*D*) resemble drug-free cultures whereby early growth is succeeded by rapid declines in density. Furthermore, there is a significant AUC decrease in penicillin-exposed cultures relative to the no-drug control one-way ANOVA: P<0.01, Fig. 1 *C* (*Inset*) and no significant change in AUC with rifampicin [Fig. 1 *D* (*Inset*)].

### Longevity Benefits Are Lost When a Ribosomal-Protection Resistance Mechanism Is Present.

We asked whether antibiotic resistance would affect the above longevity benefits. When treated with doxycycline, resistant *E. coli* should exhibit an increased growth rate during the initial growth phase, but the effect on longevity during a subsequent starvation phase is unknown. Several mechanisms confer resistance to doxycycline, including efflux (32), alterations to antibiotic structure (33) and ribosome protection (34). We therefore deployed 2 strains, *E. coli* (Wcl) and *E. coli* (GB(c)) in the 28 d TGD protocol (Methods B), namely, GB(c) and Wcl which both originate from the parental strain *E. coli* (MC4100). Importantly, GB(c) resists doxycycline using a ribosomal protection protein expressed via tet36 on a nontransmissible plasmid (35) whereas Wcl lacks tet36 resistance and therefore acts as a control. GB(c)’s protection proteins bind the ribosome and create a conformational change that dislodges bound doxycycline, freeing it for tRNA binding and allowing protein synthesis to proceed.

Wcl and GB(c) populations were exposed to doxycycline (0.2 and 0.4 mg/L) at inoculation or left drug-free for 28 d without fresh nutrients nor antibiotic (3 replicates). We observed no longevity benefit in doxycycline-exposed GB(c) cultures relative to drug-free populations and, instead, we observed reduced population densities beyond 24 h during the starvation phase (Fig. 1*E*). Indeed, GB(c) exposed to 0.2 and 0.4 mg/L doxycycline have a ∼66% and ∼97% (CFU per mL) reduction from their mean observed peak density by day 10, respectively, compared to a mean ∼65% reduction in drug-free. Thus GB(c) experiences statistically significant long-term density reductions in doxycycline (one-way ANOVA, P<0.0001, measured using AUC) relative to drug-free conditions (Fig. 1 *E*, *inset*), i.e., GB(c) accrues no longevity benefits from doxycycline. Conversely, and as expected, Wcl does derive longevity benefits from doxycycline (Fig. 1*F*).

The MIC (minimal inhibitory concentration) of doxycycline for GB(c) far exceeds that of MG1655 (*SI Appendix*, Figs. S1 and S6) so we determined density dynamics of GB(c) at higher concentrations of doxycycline, up to the IC${}_{90}$ (24 and 32 mg/L, Methods B). Data mirror those of lower doses: GB(c) accrues no benefit to population longevity from doxycycline (*SI Appendix*, Fig. S7).

### Genomic Polymorphisms Arise During Starvation with Doxycycline.

Nutrient exhaustion and aging can induce genomic diversification in bacterial populations (36, 37) so we asked whether doxycycline-associated longevity benefits might have genomic signatures. We therefore performed Illumina whole genome sequencing (WGS) during a 21 d TGD protocol whereby different populations of MG1655 were either left doxycycline-free or initially exposed to doxycycline (0.2 and 0.4 mg/L, 4 replicates) in M9CAA (Methods F; coverages displayed in *SI Appendix*, Table S1). No nutrients nor antibiotics were added at any later time and samples were removed periodically for DNA sequencing and to measure population densities (*SI Appendix*, Fig. S8).

Over 400 SNPs were observed across all drug conditions and seven time points. Most SNPs were dynamically transient and observed at low frequency, possibly due to low levels of cell turnover. More novel high-frequency SNPs were identified in doxycycline conditions than drug-free (6 in drug-free, 19 in 0.2 mg/L doxycycline, and 13 in 0.4 mg/L doxycycline), with the majority being nonsynonymous. However, the ratios of nonsynonymous to synonymous substitutions, written dN/dS, provide no evidence of differential selection on protein-coding regions between doxycycline and drug-free conditions (*SI Appendix*, Fig. S9 shows dN/dS data).

We expected polymorphisms related to the ROS response, and notably, multiple SNPs were identified in *rsxC* which helps maintain a reduced state of *soxS*, a transcriptional activator of the superoxide response regulon (38). Mutations above a 10% frequency in the population were identified across all conditions in *ftsE*, a gene involved in cell division associated with the acid stress response (39) and *cyoB*, a component of the aerobic respiratory chain.

**Fig. 2.**
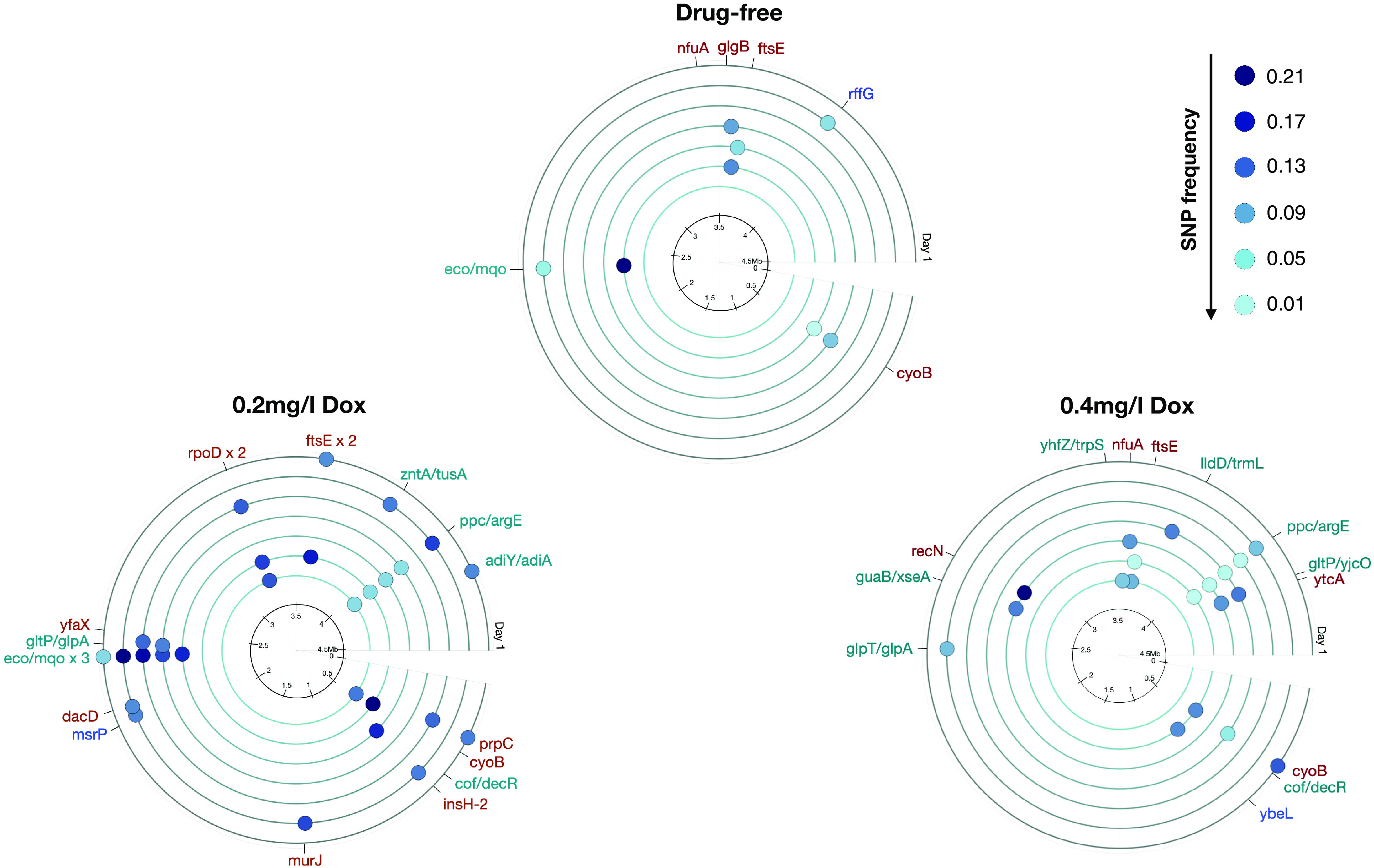
Single nucleotide polymorphisms (SNPs) identified in nutrient-starved cultures at frequencies above 10% within each population. These SNPs were identified at different time points during 21 d of nutrient starvation in drug-free and doxycycline-exposed (0.2 and 0.4 mg/L, Methods F) MG1655 cultures. SNPs in red are nonsynonymous, blue are synonymous, and green are intergenic. The outer circle represents day 1, through to days 3, 7, 10, 14, 17, and finally day 21 on the inner circle. The black inner ring indicates genome position and dot color indicates SNP frequency. These indicate that many SNPs were transient and once initially observed above 10%, they could, thereafter, recede below that value. Genes with SNPs in all conditions were involved in cell division (*ftsE*) and the aerobic respiratory chain (*cyoB*). SNPs observed only in populations treated with 0.2 mg/L doxycycline include *rpoD*, an RNA polymerase gene. Populations exposed to 0.4 mg/L doxycycline harbored SNPs in the DNA repair gene *recN*. A complete list of SNPs across all conditions is given in *SI Appendix*, Table S2, and population density dynamics are shown in *SI Appendix*, Fig. S8; the latter are consistent with the doxycycline-enhanced longevity phenotypes observed in Fig. 1*A*. Note: In any condition, 21% was the highest frequency at which a novel SNP was observed. For raw coverage levels corresponding to these SNP frequencies, see *SI Appendix*, Table S1.

Some polymorphisms above 10% frequency were unique to doxycycline treatments: Populations at 0.2 mg/L doxycycline harbored multiple SNPs in *rpoD*, an RNA polymerase implicated in growth on minimal media (40). Indeed, mutations in RNA polymerase core enzyme have been observed at high frequencies in starved bacteria (36). Populations at 0.4 mg/L doxycycline possessed SNPs in *recN*, a gene involved in the repair of DNA double-strand breaks that arise during starvation (41). Fig. 2 and *SI Appendix*, Table S2 summarize all SNPs identified at a frequency above 10%. When such a SNP is identified, it is also displayed in Fig. 2 at later time points, even if its frequency is then below 10%.

### Doxycycline Improves Growth on Cell Debris in Supernatant.

Recall that doxycycline and erythromycin-exposed populations of MG1655 experience oscillatory growth phases (Fig. 1 *A* and *B*) despite the exhaustion of glucose (*SI Appendix*, Fig. S4). Cell debris and excreted metabolites become resources during starvation (42, 43), whereby the death of subpopulations can allow other cells to proliferate. So, where antibiotics promote population densities, might this involve the exploitation of cell debris?

To find out, we cultured MG1655 in spent supernatant and determined differences in growth with the addition of doxycycline (Methods C). MG1655 was first cultured for 48 h in M9CAA to ensure complete glucose exhaustion (*SI Appendix*, Fig. S4), spent supernatant was then isolated and filter sterilized to remove cells, leaving only debris and organic compounds. Fresh MG1655 was then reinoculated into the supernatant supplemented with either 0.2 mg/L of doxycycline, 2 mg/mL of glucose, or both, in triplicate. If doxycycline does improve the exploitation of spent supernatant, we anticipate either faster growth or higher population densities in doxycycline-exposed cultures than doxycycline-free.

**Fig. 3.**
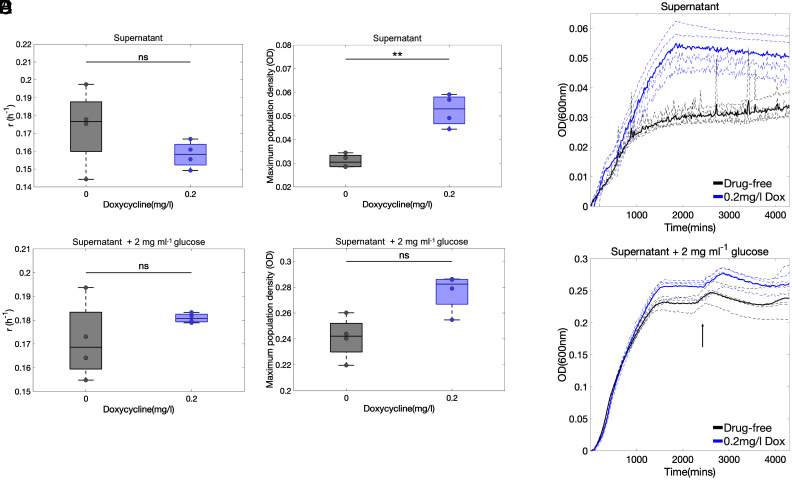
Doxycycline enhances growth on the debris in supernatant. “Oscillatory” growth–death phases observed in doxycycline and erythromycin-exposed MG1655 populations after the exhaustion of glucose (Fig. 1) suggest alternative nutrients may be supporting growth, possibly from cell debris. To test this MG1655 was grown in spent supernatant, either supplemented with 0.2 mg/L doxycycline, 2 mg/mL glucose, both, or else left with no addition to the supernatant (Methods C). (*A*) In glucose-free supernatant, growth rate (r) was not significantly altered by the addition of doxycycline; however, (*B*) the maximal observed population density (K) was significantly higher (*t*-test, P<0.012) than in drug-free conditions. This suggests doxycycline improves the exploitation of organic compounds found in spent supernatant because doxycycline-treated cultures achieve a greater population density than drug-free, and the nutrient source following glucose exhaustion is likely to come from cell debris. Neither r (*C*) nor K (*D*) differed significantly between doxycycline and drug-free populations in glucose-supplemented supernatant. (*E*) The increased population density in doxycycline-treated supernatant is evident from these 72-h growth curves. (*F*) Within glucose-supplemented supernatant, there is some evidence of a diauxic shift (indicated by an arrow) both in doxycycline and drug-free cultures, indicative of the presence of a secondary carbon source. (In *A*–*D*, data medians and first and third quartiles are shown in boxplots; in *E* and *F*, mean growth data are solid lines; replicates are dashed lines, n=4 throughout).

There was no significant difference in growth rates (hereafter denoted “r”) of different cultures, whether doxycycline-exposed or not (Fig. 3*A*), but we did observe a significant increase in maximum population densities (hereafter denoted “K”) in doxycycline-exposed populations (P<0.012, 2-sample, 2-sided *t*-test, Fig. 3*B*). Cultures supplemented with glucose had no significant doxycycline-mediated difference either in r or K (Fig. 3 *C* and *D*) and there was some evidence of diauxic growth in doxycycline-exposed and doxycycline-free conditions (Fig. 3*F*).

### Carbon Metabolism Gene Expression Changes due to Doxycycline.

Diauxic growth (44) may be observed when microbes use different limiting sugars (45) in 2 phases of growth whereby use of the second follows exhaustion of the first. The diauxy, we observe is consistent with a second resource present in the supernatant (other than the glucose in Method C, Table 1), which could be acetate produced during growth on excess glucose (46). Acetate is produced by overflow metabolism (47, 48) when fermentation is used rather than respiration and, we note, the glyoxylate shunt bypasses the TCA cycle and uses fatty acids and acetate, both of which may be present in spent supernatant (49, 50).

**Table 1.** The conditions used in the supernatant assay

No. replicates	Conditions
4	Supernatant
4	Supernatant + 0.2 mg/L doxycycline
4	Supernatant + 2 mg/mL glucose
4	Supernatant + 2 mg/mL glucose + 0.2 mg/L doxycycline
4	M9CAA
4	M9CAA + 0.2 mg/L doxycycline

A higher K achieved by doxycycline-exposed cultures, given glucose exhaustion, implies increased metabolic efficiency. This could arise from differential utilization of the above pathways (46) if doxycycline or erythromycin promoted the use of more ATP-efficient respiration when slowing growth relative to less ATP-efficient fermentation. If true, this could provide a basis for the higher K observed during 28 d cultures following doxycycline or erythromycin exposure (Fig. 1 *A* and *B*). This idea is broadly consistent with the observation that translation-targeting antibiotics can mediate metabolism (51, 52), so too the observation that carbon metabolism itself mediates antibiotic resistance (53).

To better understand the pathways doxycycline mediates, we used an MG1655 library with GFP-tagged promoters of over 1,900 genes (54) to examine strains tagged in glycolysis, the TCA cycle and acetate biosynthesis/metabolism gene promoters (*SI Appendix*, Table S3). We measured their GFP expression, a proxy for promoter expression, quantifying the latter in the presence (0.4 mg/L) and absence of doxycycline (Methods D).

**Fig. 4.**
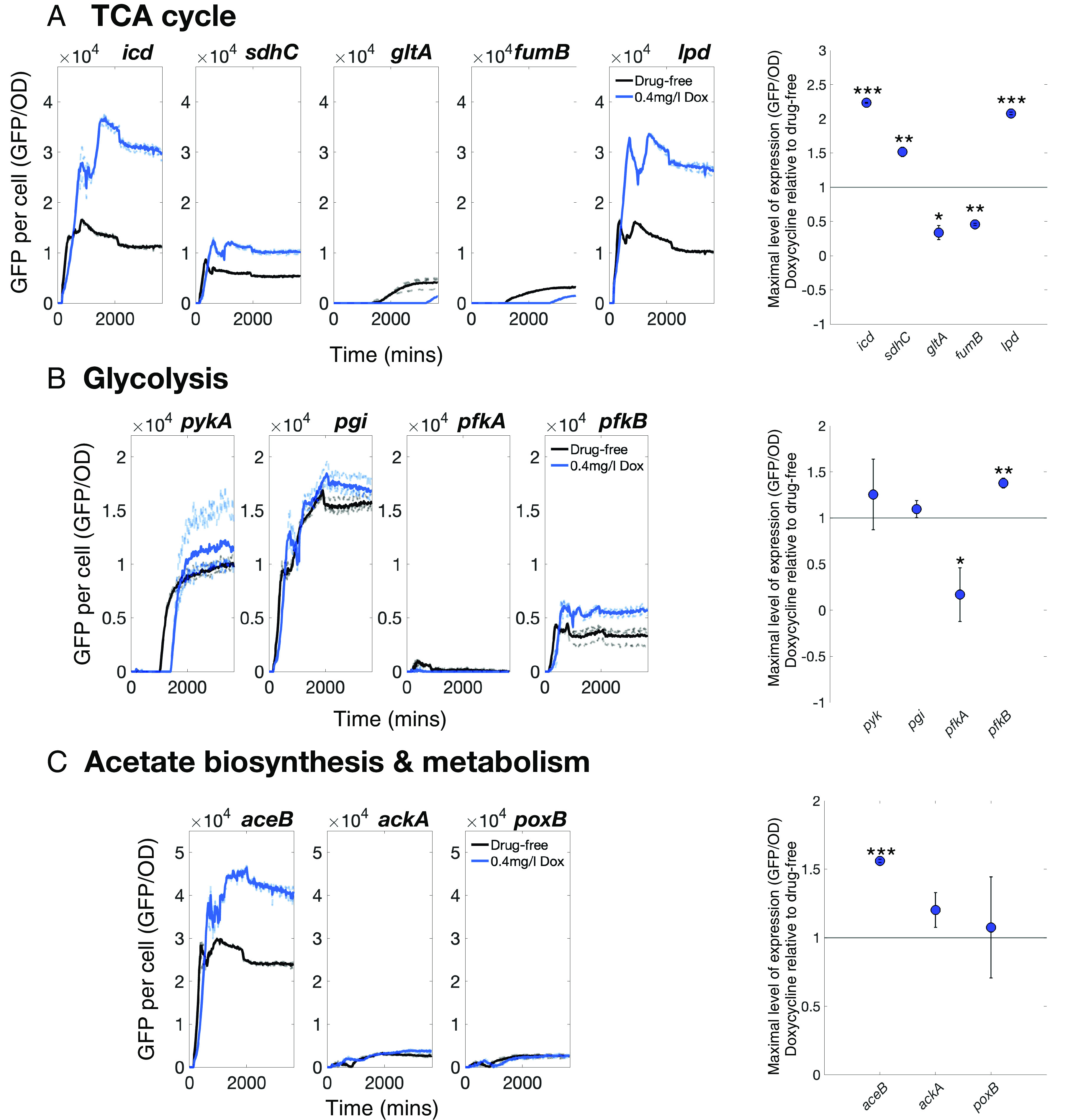
Doxycycline mediates metabolic expression in an *E. coli* library with GFP-labeled promoters. We sought doxycycline-mediated changes to metabolism that could provide a basis for observed population density and longevity benefits. For this, we used a GFP-tagged promoter library of MG1655 (Methods D). Expression kinetics were measured as GFP per OD, with GFP expression a proxy for promoter expression, based on promoters that regulate the expression of (*A*) TCA cycle, (*B*) glycolytic, and (*C*) acetate biosynthesis/metabolism genes in 0.4mg/L doxycycline and drug-free conditions. The mean is a solid line and replicates are dashed lines (*n* = 3; raw OD and GFP measurements are shown for each gene in *SI Appendix*, Figs. S14–S16). (*A*) Doxycycline induces changes in the promoter expression of all TCA cycle genes measured here. (*B*) A promoter for the glycolysis gene, *pfkA*, is down-regulated by doxycycline, although the promoter expression for this gene is low in drug-free conditions, in contrast to the others measured. Both the promoters for the (*B*) glycolysis gene *pfkB* and (*C*) acetate metabolism/glyoxylate shunt gene *aceB* are significantly up-regulated by doxycycline. The glyoxylate shunt is required for the metabolism of acetate and fatty acids, consequently the up-regulation of *aceB* could improve the use of cell debris as a nutrient source in doxycycline-treated conditions. The rightmost column shows whether doxycycline increases or decreases promoter expression based on maximal differences relative to drug-free conditions, dots are means, bars are 95% CIs (2-sample, 2-sided *t*-test, *P< 0.05, **P< 0.01, ***P< 0.001, n=3).

Among TCA genes, promoters for *icd*, *lpd*, and *sdhC* were up-regulated in the presence of doxycycline (Fig. 4*A*) with a ∼2.5x higher maximum expression of both *lpd* and *icd* relative to drug-free cultures and a ∼1.5x higher maximum expression of *sdhC*. Conversely, promoters for TCA genes *fumB* and *gltA* were both down-regulated by doxycycline (Fig. 4*A*). The promoter for glycolytic gene *pfkB* was significantly up-regulated and *pfkA* significantly down-regulated by doxycycline, though the latter was not highly expressed (Fig. 4*B*). We also performed this assay for acetate biosynthesis gene promoters that regulate the expression of *ackA* and *poxB*, and *aceB*, a glyoxylate gene. Neither *ackA* or *poxB* saw a significant change in expression due to doxycycline (Fig. 4*C*). However, the *aceB* promoter is up-regulated by doxycycline with ∼1.6x higher maximum expression relative to the drug-free control (Fig. 4*C*). Thus doxycycline affects carbon metabolism although it remains unclear whether it increases biomass production efficiency by altering the expression of metabolic genes.

### ROS-Response Mutants Exhibit Growth Benefits from Doxycycline.

As doxycycline is an antioxidant (6, 25), we anticipated *E. coli* lacking genes in the response to ROS could accrue benefits from doxycycline. To assess this, we determined how doxycycline mediates population growth using the Keio library of *E. coli* (BW25113) mutants with each nonessential gene knocked out and replaced with a kanamycin resistance cassette (55). We measured the growth of 10 knockouts involved in the ROS response (*soxS, soxR, sodA, sodB, sodC, perR, ahpC, katE, katG* and *oxyR*) and of the general stress response sigma factor, *rpoS*. Each knockout strain was cultured for 48 h in doxycycline and doxycycline-free conditions and growth was quantified (Methods E).

**Fig. 5.**
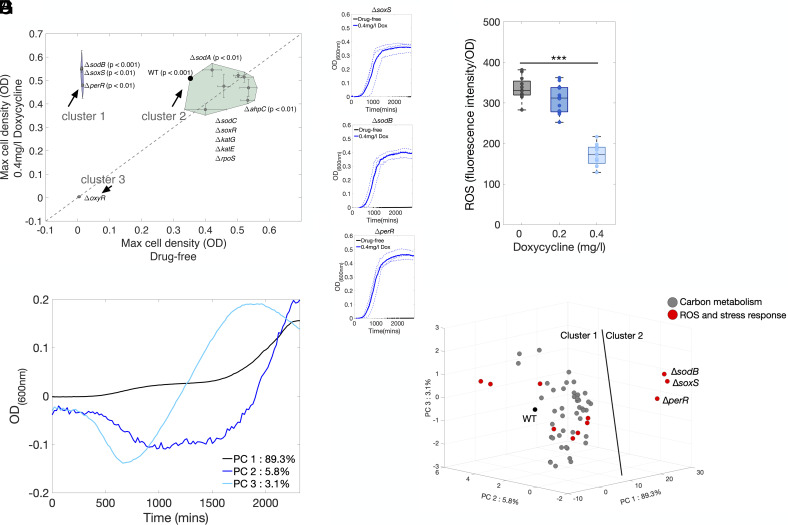
Does doxycycline benefit the growth of ROS- and metabolism-related knockouts? (*A*) To test whether doxycycline improves the growth of Keio knockouts involved in ROS and stress responses, maximum cell densities with and without doxycycline after 48 h of growth are plotted for 12 strains (Methods E, n=3, mean ± SE). 3 clusters of strains are shown surrounded by convex hulls whereby in one (cluster 1, containing Δ*soxS*, Δ*sodB* and Δ*perR*), doxycycline is needed for strains to grow. Cluster 2 has 2 strains whose maximal density is larger when doxycycline is present (*t*-test; green convex hull): the Keio parent (WT) and Δ*sodA*. Cluster 3 contains one knockout, Δ*oxyR*, that did not grow at all. (*B*–*D*) Raw growth data for cluster 1 strains in *A* (mean shown as a solid line, replicates shown as dashed lines, *n* = 3). (*E*) ROS concentrations were estimated using a ROS-sensitive fluorescent dye for 24 h of *E. coli* MG1655 growth in drug-free and doxycycline-treated conditions (*SI Appendix*, *Materials and Methods*): 0.4 mg/L doxycycline led to significantly lower fluorescence than drug-free conditions (one-way ANOVA, F${}_{\left(2,33\right)}$ = 105.04, P<0.001 with post hoc Tukey; asterisks represent the P value: ***P<0.001, *n* = 12). (*F*) We measured growth of 48 Keio knockouts involved in carbon metabolism alongside 11 strains involved in ROS and stress protection (strains in *SI Appendix*, Table S4, growth data in *SI Appendix*, Figs. S10 and S11, *n* = 1). Density differences of ROS- and metabolic-related Keio strains cultured with and without doxycycline underwent a PCA analysis that represents those differences mathematically as a linear combination of vectors PC 1, PC 2, and PC 3 that account for 98.2% of variation in data. The first PCA vector, PC 1, does not change sign at any time, commensurate with doxycycline reducing population density through all phases of growth for most strains. However, the second and third PCA vectors, PC 2 and PC 3, both exhibit a rate–yield tradeoff (RYTO) whereby reductions in exponential phase growth, i.e., PC 2 and 3 have negative y-axis data around 1,000 min (16 h) come with increases in later phase densities (i.e., positive y-axis data at 2,000 mins). (*G*) Data were projected onto the first 3 PCA dimensions and K-means clustering reveals 2 clusters: one contains all strains tested apart from ROS knockouts Δ*soxS*, Δ*sodB* and Δ*perR*.

Interestingly, 3 knockout strains, *ΔsoxS, ΔsodB* and *ΔperR* have an “extreme benefit” from this treatment in the sense that they do not grow in doxycycline-free M9CAA but they do grow when doxycycline is present (Fig. 5*A*, cluster 1; Fig. 5 *B*–*D* shows growth data). Furthermore, some knockouts benefit by having higher population densities in doxycycline (Fig. 5*A*, data above the line “x=y”). One strain, Δ*oxyR*, was unable to grow with, or without, doxycycline.

The restoration of growth of strains *ΔsoxS, ΔsodB*, and *ΔperR* could be due to doxycycline’s antioxidant properties (6, 25). So, to quantify the reduction in ROS due to a doxycycline treatment, MG1655 was cultured for 24 h in doxycycline-exposed (0.2 and 0.4 mg/mL, 12 replicates) and doxycycline-free conditions using a ROS-sensitive dye (*SI Appendix*, *Materials and Methods*). We observed a significantly lower level of fluorescence, a proxy for ROS concentration, in the 0.4 mg/L doxycycline culture (Fig. 5*E*, one-way ANOVA, P<0.001), consistent with lower ROS concentrations in doxycycline-treated populations. This could be explained both by the scavenging of ROS by doxycycline and the reduction in growth rate due to doxycycline.

### The Growth Rate–Yield Tradeoff Mediated by Doxycycline.

Having demonstrated doxycycline mediates both carbon metabolism and ROS-related effects on growth, we reasoned it should effect the rate–yield tradeoff (RYTO), this is the idea that slower growth accompanies more efficient metabolism (56). To investigate this we assayed 59 Keio strains involved in central carbon metabolism, *rpoS* and ROS-related changes (*SI Appendix*, Table S4) to quantify changes in growth attributable to doxycycline. We then performed principle component analysis (PCA) on the entirety of growth data from both doxycycline-free and doxycycline treatments (at 0.4 mg/L, Methods E; *SI Appendix*, Figs. S10 and S11), as follows. After subtracting doxycycline-treated growth data from its drug-free counterpart, we implemented PCA on the differences of all strains tested and applied K-means clustering to the first 3 PCA dimensions (accounting for 98.2% of the variation in the data).

The first PCA vector, PC1, accounts for 89.3% of that variation. As PC1 does not change sign, it captures how most of these doxycycline-treated populations are typically reduced monotonically throughout the culture period relative to their drug-free counterpart (i.e., doxycycline impairs all phases of growth in PC1, Fig. 5*F*). However, the next PCA vectors reveal 2 subtly different RYTOs due to doxycycline: because they change sign, PC2 (5.8% variation) and PC3 (3.1% variation) capture decreases in exponential phase growth that coincide with an increase in density during stationary phase (Fig. 5*F*).

The relative strengths of the RYTO are quantified by the second and third PCA coefficients (Fig. 5*G*) because PC1 exhibits no RYTO but PC2 and PC3 do. Scatterplots of the first 3 PCA coefficients reveal 2 evident clusters separated by a plane with the majority of strains in cluster 1 and just 3 strains in cluster 2: Δ*soxS*, Δ*sodB* and Δ*perR* (Fig. 5*G*). This is unsurprising because these 3 have the “extreme benefit” from doxycycline whereby they only grow in its presence.

In summary, this analysis shows that i) doxycycline need not benefit the growth of all metabolism and ROS-related Keio mutants (*SI Appendix*, Table S4), but it does benefit some. ii) Different Keio strains can exhibit rate–yield tradeoffs of different strengths when treated with doxycycline. iii) Doxycycline benefits are consistent with ROS scavenging which compensates for the loss of stress response genes to the extent that doxycycline can restore growth that is otherwise absent.

### Are Phenotypic Changes due to Doxycycline Correlated among Keio Strains?.

**Fig. 6.**
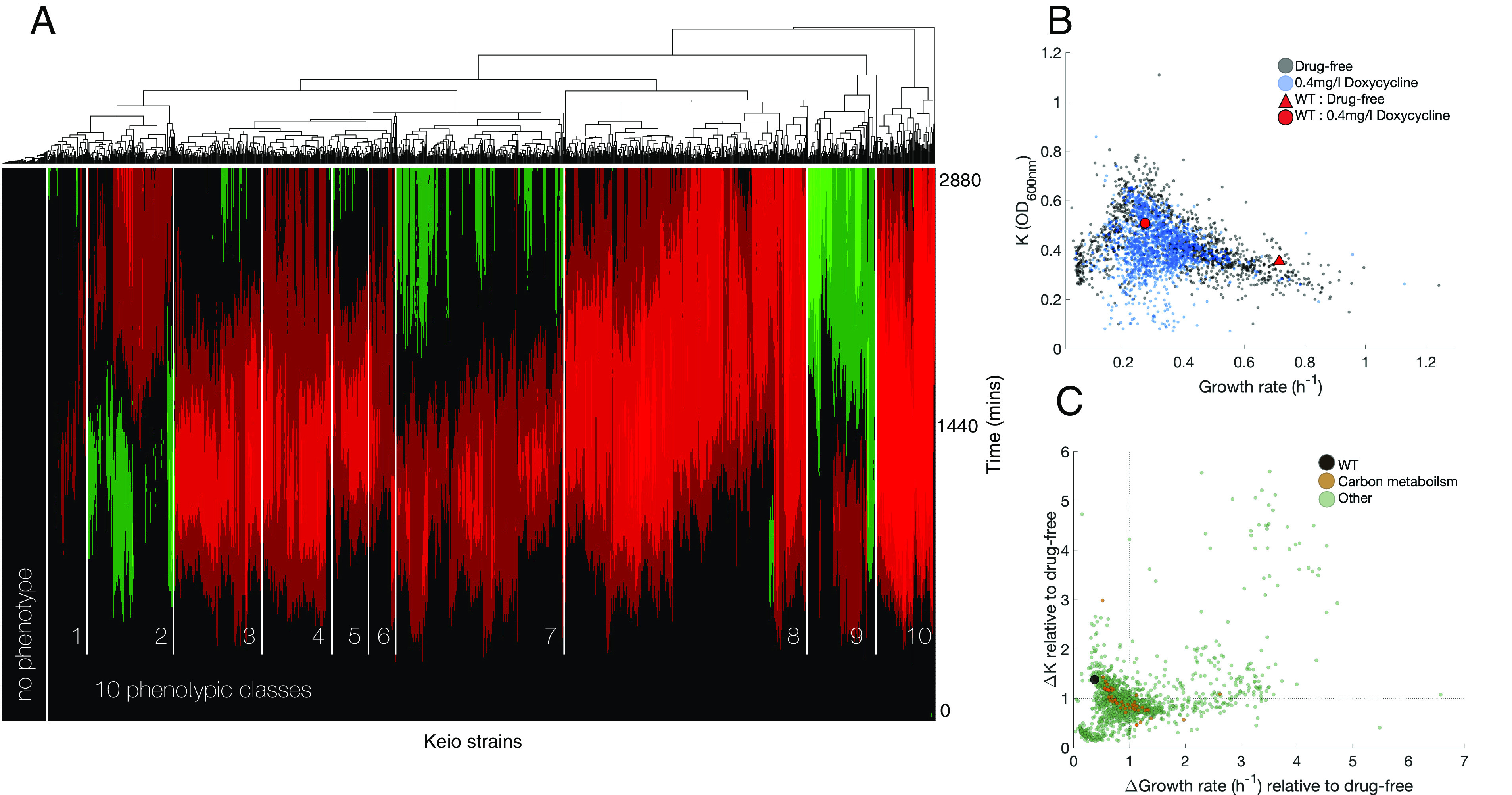
Doxycycline induces a range of phenotypic changes among Keio mutants, including r and K benefits. (*A*) Hierarchical clustering of 48-h growth data from 1,522 Keio strains in doxycycline (0.4 mg/L, IC${}_{50}$ of Keio parent BW25113 (WT here)) relative to drug-free growth reveals a range of phenotypes across different genetic backgrounds (Methods E). Strains cluster into approximately 10 phenotypic classes where green indicates OD is greater in doxycycline conditions relative to drug-free, red indicates it is lower, black indicates it is the same: thus, in red regions, doxycycline acts as an antibiotic, and in the green regions, it benefits a phase of growth. These data contain 92 strains within the entirely red regions of phenotypic classes 8 and 10 that we deem “hypersensitive” to doxycycline as they only grow in the absence of doxycycline, not its presence. The class “no phenotype” occurs for strains where doxycycline has little effect on any of the growth phases. (*B*) Scatterplot of growth rate (r) and the maximum observed population size (K) for these strains in the presence and absence of doxycycline: A between-strain rate–yield tradeoff would see greater r come with lower K but this is not observed, although most strains do have reduced r but increased K relative to WT in the drug’s absence. (*C*) A scatterplot of the relative change in r between doxycycline and drug-free cultures (denoted Δr and ΔGrowth rate) and the analogous relative change in K (denoted ΔK) is shown: 748/1,522 strains benefit from doxycycline because either i) ΔK>1, ii) Δr>1 or else both (i) and (ii) are satisfied (i.e., dual benefits). Features of note: 1) orange circles: these show r and K changes in response to doxycycline for metabolic knockouts and the change in K is smaller than WT for most of these. 2) Doxycycline reduces K (where ΔK<1) for many strains but increases it for some. 3) Most strains have larger Δr than WT, most also have smaller ΔK. Thus we observe a range of effects of doxycycline on the 2-dimensional (r,K) phenotype of the Keio library, some beneficial and some not. (For growth data supporting this, see *SI Appendix*, Figs. S17 and S18).

We sought to determine how doxycycline affects different phases of growth in a broader range of genetic perturbations for which we conducted growth assays using over 1,500 Keio strains with and without doxycycline (Methods E, at the parental strain IC${}_{50}$). After checking density data (OD) were positive at all times and sifting potential filamentation (*SI Appendix*, *Materials and Methods*), we applied hierarchal clustering to the difference in OD between drug and no-drug conditions. This identified around 10 phenotypic clusters with similar doxycycline-related changes relative to the Keio parent in different phases of growth (Fig. 6*A*). These 10 have similar growth patterns whereby the effects of doxycycline on lag, exponential, and stationary phases correlate, highlighting how the drug has varied sets of inhibitory and beneficial effects that occur in genetic clusters.

Using these data, we asked whether doxycycline-induced r and K benefits would be correlated: If r increases, does K increase? In summary, no. Distributions of drug-free and doxycycline-treated r and K phenotypes overlap significantly and so one cannot say Keio strains in drug-treated conditions tend to grow slower or else to higher densities (Fig. 6*B*). There is not a monotonic reduction of r and K due to doxycycline but, instead, a wide distribution of changes whereby 748 of the strains assayed benefit from doxycycline in either r or K, including the Keio parent (WT in Fig. 6*C* and *SI Appendix*, S12 *A*–*C*).

Let us summarize mechanisms associated with r and K improvements due to doxycycline: 446 Keio strains benefited through increased K, a property enriched in the following COG terms (57), tested via Fisher’s exact test: F (nucleotide metabolism and transport, P<0.05), K (transcription, P<0.001) and T (signal transduction, P<0.001). Growth rate, r, improved, in 460 strains with enrichment for COG terms F (P<0.01), 206 strains exhibited benefits in both K and r and these are enriched in the COG term E (amino acid transport and conversion, P<0.001).

Now, 92 Keio strains experienced “hypersensitivity” to doxycycline whereby they did not grow in a doxycycline treatment at parental IC${}_{50}$, thus these mutants’ MICs are below that IC${}_{50}$. Enriched genes for this are COG terms C (energy production and conversion, P<0.05), I (lipid metabolism, P<0.05), and Q (secondary structure P<0.05; *SI Appendix*, Fig. S12*D*). Thus, the Keio library exhibits the interesting feature that any strain differs in at most 2 loci from another Keio strain that either derives benefits from doxycycline, or else is hypersensitive to it.

### Below Wild-Type rrn Operon Copy Number Maximizes Population Longevity.

It is important to ascertain how ribosomal availability mediates antibiotic-related benefits for it is plausible they accrue from changes in the number of functional ribosomes, a property of cells that can be controlled with the antibiotics we use. Ribosome synthesis affects metabolic efficiency and growth rate (58, 59) and bacterial strains with different numbers of rRNA operons (*rrn*) have different growth rates (60), but, we ask, do different numbers of operons lead to different rates of population decline during starvation? To address this, we use 6 strains of *E. coli* with different numbers of *rrn* operons (61) relative to their parent, MG1655 (denoted WT) which has 7. These 6 strains have 1 to 6 *rrn* operons removed, decreasing the production of functional ribosomes with each deletion in a nonlinear manner, nevertheless, the removal of *rrn* operons is known to reduce ribosome availability (61).

**Fig. 7.**
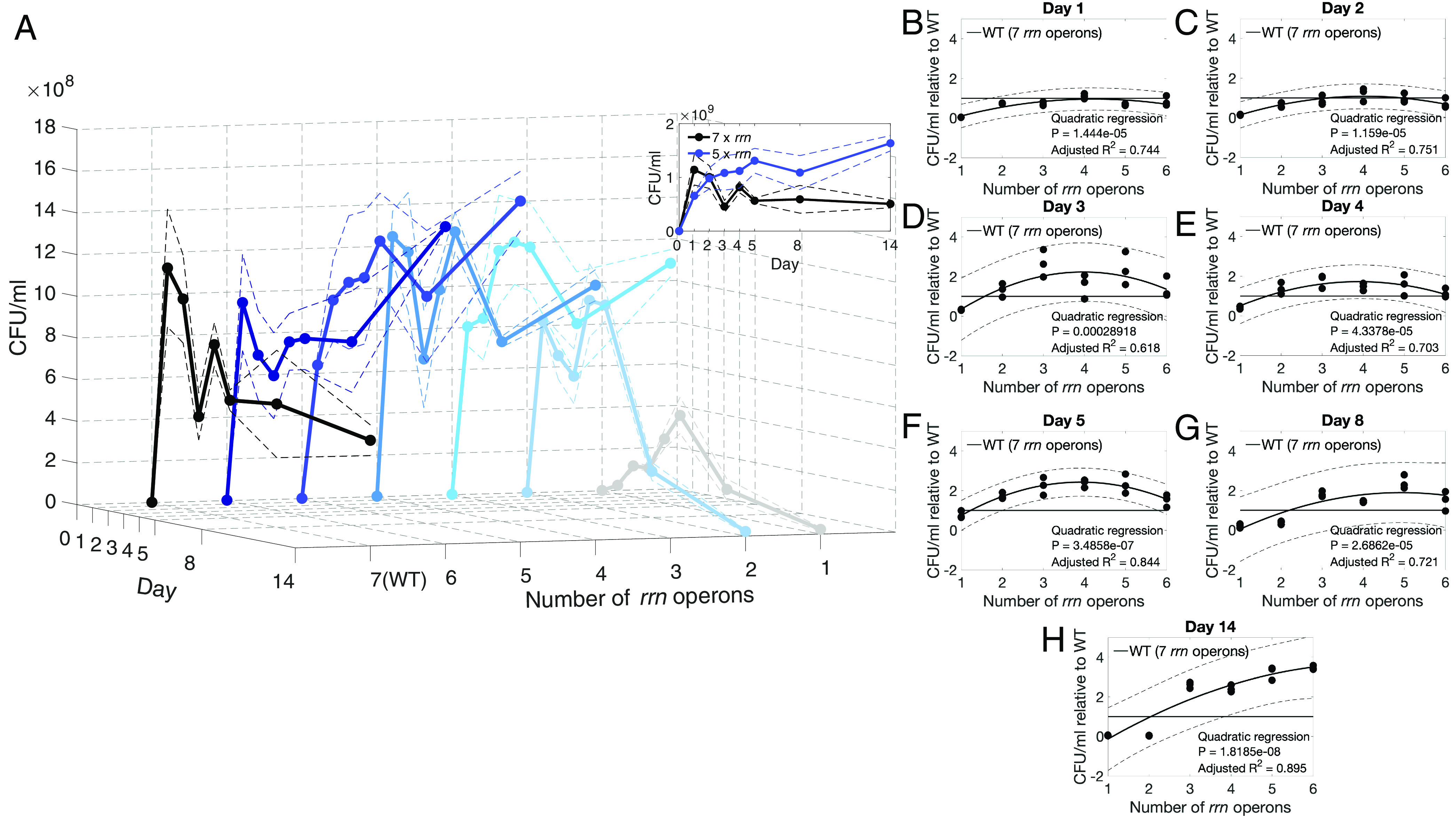
An intermediate number of *rrn* operons can be optimal for population longevity. The *E. coli* (Δ*rrn*) strains have different numbers of ribosomal RNA operons, 7 (the most here) are found in the MG1655 strain denoted WT. (*A*) Cultures of these strains lasting 14 d (Methods G) show an intermediate number of those operons is best at maintaining population density (CFU/mL on the z-axis): the WT grows and declines rapidly after day 1 whereas strains with 3 to 6 *rrn* operons have greater population densities at later times. The strain with 5 operons has a continual rise in population density over the 14 d, above WT (inset). (Solid line is mean ± 95% CIs shown as dashed lines, n=3.) (*B*–*H*) Population densities (CFU/mL) relative to the WT (y-axis) are shown as a function of *rrn* operon number (x-axis) with different subplots for different days. A value of 1 on the y-axis represents a population density equal to that of the WT on the stated day. Both linear and quadratic regressions were determined with the best fit according to the adjusted $R^{2}$ value shown as solid lines ± estimated 95% CIs displayed as dashed lines (n=3). Note how the fitted polynomial is typically maximized above 1 at an intermediate number of ribosomal RNA operons beyond day 2, meaning the WT is not optimal in terms of maximizing population density up to that day, rather some other strain with fewer *rrn* operons is, ranging from 3 to 6 operons on day 14.

All 7 strains were cultured for 14 d in M9CAA without the further addition of nutrients, no antibiotic was used at any point (Methods G). As expected, the population density of WT declined after day 1 (Fig. 7*A*, black line) but the decline is either delayed or not observed for the perturbed *rrn* strains (blue lines). The strain with 5 *rrn* operons, for example, increases in population density for 14 d (Fig. 7*A*, inset blue line) whereas the strain with 1 *rrn* operon grows poorly (Fig. 7*A*, gray line).

Consistent with expectations that different numbers of operons are optimal for different resource availabilities (59, 62), the optimal number of *rrn* operons that achieves the greatest population density depends on assay duration. For instance, there is no significant difference between 6 of the strains’ population densities on day 1 (Fig. 7*B*). By day 5 the WT does not maximize population density, rather it performs as poorly as the strain with 1 *rrn* operon, thus an intermediate number of operons is now optimal in terms of maximizing population density (Fig. 7*F*). By day 14, 6 operons are optimal in this sense (Fig. 7*H*). This suggests that while 4 to 7 *rrn* operons are optimal for the initial stages of nutrient-rich growth, a lower number, 3 to 6 *rrn* operons, have better longevity properties. Thus, ribosomal content mediates population longevity whether, or not, an antibiotic is present in the extracellular environment.

## Discussion

We were motivated by the idea that most microbial cells reside closer to kin than to foe in spatially structured environments, thus antibiotics emanating from producer cells might exert beneficial effects on kin before they encounter more distant foe. We therefore sought conditions under which antibiotics can benefit bacteria, noting that ribosome-binding antibiotics are candidates for this due to their interactions with ROS. We found several benefits of doxycycline, including the decrease in ROS, likely caused by slowed growth and the scavenging of ROS by doxycycline whereby, as a result, doxycycline benefits both the growth efficiency and longevity of *E. coli* populations.

Longevity benefits are not universal among *E. coli* strains because resistance by ribosomal protection removes them. This physical barrier highlights a key property: doxycycline must physically reach the ribosome for these benefits to accrue, it is not sufficient that the antibiotics merely interact with ROS. Indeed, otherwise, this longevity benefit would be observed in *E. coli* that possess ribosome-protection mechanisms, but it is not (Fig. 1 *E* and *F*). Other resistance mechanisms that reduce the intracellular concentration of antibiotic, resulting in fewer antibiotic-ribosome interactions, such as the increased expression of efflux pumps, may also abolish longevity benefits but we have not tested this.

We had another motivation too: the rate–yield tradeoff (RYTO) (56, 60). When bacteria exhibit a RYTO, slower growth is more efficient and populations accrue larger densities as a result. The response to ROS must mediate this tradeoff because population size, a key variable for quantifying growth efficiency, will increase when the death rate is reduced because ROS concentration reduces. Little is known about how antibiotics, or resistance, mediates this but, as a corollary to the RYTO, we suspected that slow growth due to antibiotics could lead to more efficient growth and our data demonstrates this for doxycycline. Moreover, larger population sizes observed following drug exposure, together with reductions in ROS abundance, are two reasons why drug-exposed populations exhibit increased longevity.

Other mechanisms contribute to these benefits, for instance, the observation of greater population densities cultured on supernatant in the presence of doxycycline (Fig. 3) versus lower population densities in its absence is indicative of doxycycline promoting either efficient nutrient acquisition or utilization when a primary carbon source is spent.

We used the Keio collection to determine doxycycline-related benefits in other life history traits, depending on the genetic background. For instance, doxycycline can increase the growth rate and population density of some strains and it can restore the growth of certain ROS-related lethal gene knockouts whereby those knockout strains do not grow unless doxycycline is present (Fig. 5). We deemed some Keio strains hypersensitive to doxycycline and, taken overall, doxycycline has detrimental effects to most strains in most phases of *E. coli* growth (red in Fig. 6*A*) but not all (green in Fig. 6*A*).

We have shown antibiotics are not needed to mediate population longevity via the ribosome, the number of rRNA operons in the *E. coli* chromosome does this too. The greatest number of rRNA operons (i.e., 7 of the *rrn* WT, MG1655) maximizes growth rate in nutrient-rich conditions (59) and, of the 6 rRNA knockout strains used here, the life-death tradeoff predicts the WT would have the greatest rate of population decline. This expectation is broadly consistent with data: the WT has the greatest population densities at 24 h, however a different optimal number, namely 5 rRNA operons, maximizes population densities beyond 24 h (Fig. 7*A*). Strains with 1 rRNA operon do grow but slowly and to the lowest densities. Strains with 2 operons grow and die relatively quickly and so a life-death tradeoff is present between some, but not all, of these strains (Fig. 7*A*). Thus, although *rrn* copy number and growth rate correlate linearly, as do *rrn* copy number and carbon use efficiency (59), population densities have nonlinear (i.e., quadratic) dependence on *rrn* copy number because intermediate numbers of operons maximize population densities at later times (Fig. 7 *B*–*H*).

We speculate this has consequences for the maintenance of antibiotic resistance polymorphisms, even in the absence of the antibiotic. To explain, consider erythromycin, an antibiotic that exhibits polymorphism in the clinic whereby some global distributions of MICs for erythromycin have both resistant (R) and susceptible (S) subpopulations in surveillance databases (63). Motivated by this, we ask, what mechanisms can maintain both S and R? We propose this mechanism can: bacteria with more rRNA operons have more drug targets per ribosome-binding antibiotic and are, therefore, less sensitive to it (64) (*SI Appendix*, Fig. S13) but these also have reduced longevity (Fig. 7*A*). Thus, if bacteria experience environments that switch between feast and famine, as a corollary, stable subpopulations with high and low numbers of *rrn* operons (R and S, respectively) could be comaintained because both are selected for in different environments, even if the antibiotic is absent. This implies too that the resistance phenotype of a population once antibiotic treatment starts, namely the relative frequency of S and R, can depend on the nutrient stresses the population experienced prior to treatment. This idea mirrors recent arguments for comaintenance made using theoretical models (65).

A technical limitation of this study arises when measuring phenotypes like population density and population mean GFP expression using spectrophotometry, as here. We have endeavored to use cell counts where practicable but this is not possible when screening large strain libraries. As a result, it is plausible that some measurements do not work as intended because, for example, of filamentation or unusual cell size characteristics that pass undetected. While we describe an algorithm for detecting some of these problematic features (*SI Appendix*, Fig. S19), we cannot be sure we have isolated them all from our analysis.

Finally, measuring the therapeutic benefit of antibiotics using short-term clinical assays may still be appropriate because pathogens facing an immune response may not survive for long and so rapid growth could well be optimal in vivo. However, in contexts where microbes endure nutrient stresses over long time periods, we argue that antibiotics can help bacteria navigate those periods, inverting the benefits of resistance in the process. So although antibiotics can behave as warfare molecules (66, 67), our data add to the evidence that they can play other roles in bacterial lifestyles (68, 69).

## Materials and Methods

### Media and Culture Conditions.

All strains were cultured in liquid M9 minimal media supplemented with 0.2% glucose (w/v) and 0.1% casamino acids (M9CAA). Prior to use, bacteria were grown in 10 ml M9CAA for 24 h at 30 ${}^{{}^{\circ}}$C.

Throughout, cell density is measured either by counting colony forming units (CFU) or by taking optical density (OD) readings (600 nm) using a Tecan Spark microplate reader. OD readings are used as a proxy for cell density and were found to correlate well with colony counts (*SI Appendix*, *Materials and Methods*). Strains used in this study are listed in the *SI Appendix*, *Materials and Methods* and *SI Appendix*, Table S6.

### A: Long-Term Culture of E. coli (MG1655)—the 28d Treatment–Growth–Death (TGD) Protocol.

Overnight cultures of *E. coli* (MG1655) were used to inoculate 30 ml of M9CAA in triplicate at an initial density of 1 ×10^6^ cells/ml, supplemented with either doxycycline (0.2 and 0.4 mg/L), erythromycin (2 and 5 mg/L), rifampicin (0.25 and 0.5 mg/L) or penicillin (0.125 and 0.25 mg/L) at concentrations that inhibit 20% (IC${}_{20}$) or 50% (IC${}_{50}$) of growth, or else left drug-free. Additional cultures containing higher concentrations of doxycycline up to the IC${}_{90}$ (0.8 and 1 mg/L) were also prepared. These cultures were incubated for 28 d at 30 ${}^{{}^{\circ}}$C and shaken at 160 rpm without the further addition of the antibiotic or nutrients. At regular intervals (days 0, 1, 3, 6, 8, 10, 16, 20, and 28), 10 μl was removed from each culture, serially diluted, and spread onto LB agar plates for colony counting. Certain antibiotics are known to induce bacterial filamentation (70, 71); however, the use of CFU measurements as a proxy for cell density mitigates against this.

### B: Long-Term Culture of E. coli (Wcl) and E.coli (GB(c)).

To measure the impact of doxycycline resistance on longevity, *E. coli* (Wcl) (doxycycline-sensitive) and *E. coli* (GB(c)) (doxycycline-resistant) were inoculated separately into 30 ml M9CAA in triplicate at an initial density of 1 × 10^6^ cells/ml and supplemented with doxycycline (0.2 and 0.4 mg/L), or left drug-free. Additional cultures containing high concentrations of doxycycline (24 and 32 mg/L) were also prepared for *E. coli* (GB(c)). These cultures were incubated for 28 d at 30 ${}^{{}^{\circ}}$C and shaken at 160 rpm without the further addition of antibiotic or nutrients. At regular intervals (days 0, 1, 3, 6, 8, 10, 16, 20, and 28), 10 μl was removed from each culture, serially diluted, and spread onto LB agar plates for the purpose of colony counting. The longevity of Wcl was compared against that of GB(c) here as both are derived from the same parent strain, MC4100.

### C: Supernatant Growth Assay.

The following protocol determined the effect of doxycycline on growth in supernatant. First, 1 × 10^6^ cells/ml of *E. coli* (MG1655) was inoculated into 30 ml M9CAA and grown at 30 °C for 48 h until complete glucose exhaustion (*SI Appendix*, Fig. S4). Cultures were then centrifuged at 4,300×g for 3 min, after which the supernatant was transferred to a fresh tube and bacterial cell pellet discarded. This centrifugation step was repeated, followed by filter sterilization using a 0.22-µm filter unit, leaving only small organic molecules within the media behind. To reinoculate the supernatant, fresh overnight cultures of MG1655 were grown in 30 ml M9CAA for 24 h and centrifuged at 1,700×g for 2 min. The supernatant was discarded, and the bacterial pellet was resuspended in 10 ml PBS and this centrifugation step was repeated a further 3 times to minimize any M9CAA present. The washed cells (1 × 10^6^ cells/ml) were inoculated into 1 ml of prepared supernatant per condition and into M9CAA controls. The conditions and number of replicates are detailed in Table 1. 150 μl of each culture was inoculated into a 96-well microplate and incubated at 30 °C. Cell density was measured as OD(600 nm) every 20 min for 72 h in a Tecan Spark microplate reader.

### D: Measurement of Promoter Activity.

To study the effects of doxycycline on the expression of different promoters, a library of *E. coli* (MG1655) reporter strains was used in which a fast-folding GFP is fused to a full-length copy of the *E.coli* promoter in a low-copy plasmid (72). This allows GFP expression to be used as a proxy for promoter expression.

The GFP promoter library was received from Horizon Discovery (Cambridge, United Kingdom) in 96-well microplates and immediately stored at −80 ${}^{{}^{\circ}}$C. Prior to use, a pin replicator was used to transfer each strain into a microplate containing 150 µl M9CAA within each well. The microplates were then incubated for 24 h at 30 ${}^{{}^{\circ}}$C.

Twelve GFP reporter strains (*SI Appendix*, Table S3) were inoculated into 150 µl of M9CAA in triplicate (either drug-free or with 0.4 mg/L doxycycline). OD (600 nm) and GFP fluorescence (485/520 nm) were measured every 20 min in a Tecan Infinite 200 Pro microplate reader. GFP measurements were normalized to the respective OD readings for each strain to obtain GFP/OD and the maximal GFP expression was defined as the maximum level of GFP fluorescence obtained per cell (GFP/OD). Background fluorescence was subtracted using the GFP measurements from a reporter strain with a promoterless vector (pUA66) supplied with the reporter library.

### E: Growth of the Keio Strains.

The Keio strain library (approximately 4,000 mutants of *E. coli* K12 (BW25113)) was received from Horizon Discovery in 96-well microplates and immediately stored at −80 ${}^{{}^{\circ}}$C. Prior to use, a pin replicator was used to transfer each culture into a microplate containing 150 µl M9CAA within each well. The microplates were then incubated for 24 h at 30 ${}^{{}^{\circ}}$C. Selected Keio strains were transferred to 150 µl M9CAA (either drug-free or with 0.4 mg/L doxycycline) within a 96-well microplate using a pin replicator and grown at 30 °C for 48 in a Tecan Spark microplate reader, with OD(600 nm) readings taken every 20 min. Some strains were suspected of filamentation and were removed from further data analysis (*SI Appendix*, *Materials and Methods*). Note: 1,528 Keio strains were screened for mutants with phenotypes of interest as single replicates, such as doxycycline-dependent growth, any subsequent strains of interest based on that screen (e.g., Keio strains related to ROS and metabolism) were then grown in triplicate as validation.

### F: Whole Genome Sequencing of Starved E. coli (MG1655) Cultures.

To determine genomic changes contributing to the benefits observed in doxycycline-exposed cultures, we sequenced genomes of *E. coli* (MG1655) populations (either drug-free or treated once with 0.2 or 0.4 mg/L doxycycline at the point of inoculation) throughout a 21 d period of nutrient starvation. Four replicates of each condition (0, 0.2, and 0.4 mg/L doxycycline) were used; the *SI Appendix*, *Materials and Methods* details the experimental protocol and data analysis.

### G: Long-Term Culture: E. coli (MG1655) Δrrn Strains.

The role of ribosome operon number on population longevity was assessed by culturing *E. coli* (MG1655) (Δrrn) knockout strains (*SI Appendix*, Table S6) in triplicate for 14 d in 30 ml of M9CAA without antibiotics. Cultures were incubated at 30 °C and shaken at 160 rpm. On days 0, 1, 2, 3, 4, 5, 8, and 14, 10 μl was removed from each culture, serially diluted, and spread onto LB agar plates for colony counting.

## Supplementary Material

Appendix 01 (PDF)

## Data Availability

Phenotypic data in all figures from the main text can be downloaded from Zenodo repository available via the link https://zenodo.org/record/8334696 (73).
