# Supplementary material for: Ribosome-binding antibiotics increase bacterial longevity and growth efficiency
Source: Proc Natl Acad Sci U S A. 2023 Sep 26;120(40):e2221507120. doi: 10.1073/pnas.2221507120 (PMC10556576; doi:10.1073/pnas.2221507120)
Supplement: Supplementary file 1 — Appendix 01 (PDF) [file pnas.2221507120.sapp.pdf]

Supporting Information for

Ribosome-Binding Antibiotics Increase Bacterial Longevity and Growth Efficiency

Emily Wood*, Hinrich Schulenburg, Philip Rosenstiel, Tobias Bergmiller, Dyan Ankrett, Ivana Gudelj, Robert Beardmore*

*** To whom correspondence should be addressed: R.B or E.W**
E-mail: R.E.Beardmore@exeter.ac.uk (R.B) or E.J.Wood@exeter.ac.uk (E.W)

This PDF file includes:

SI Materials and Methods
Figs. S1 to S20
Tables S1 to S7
SI References

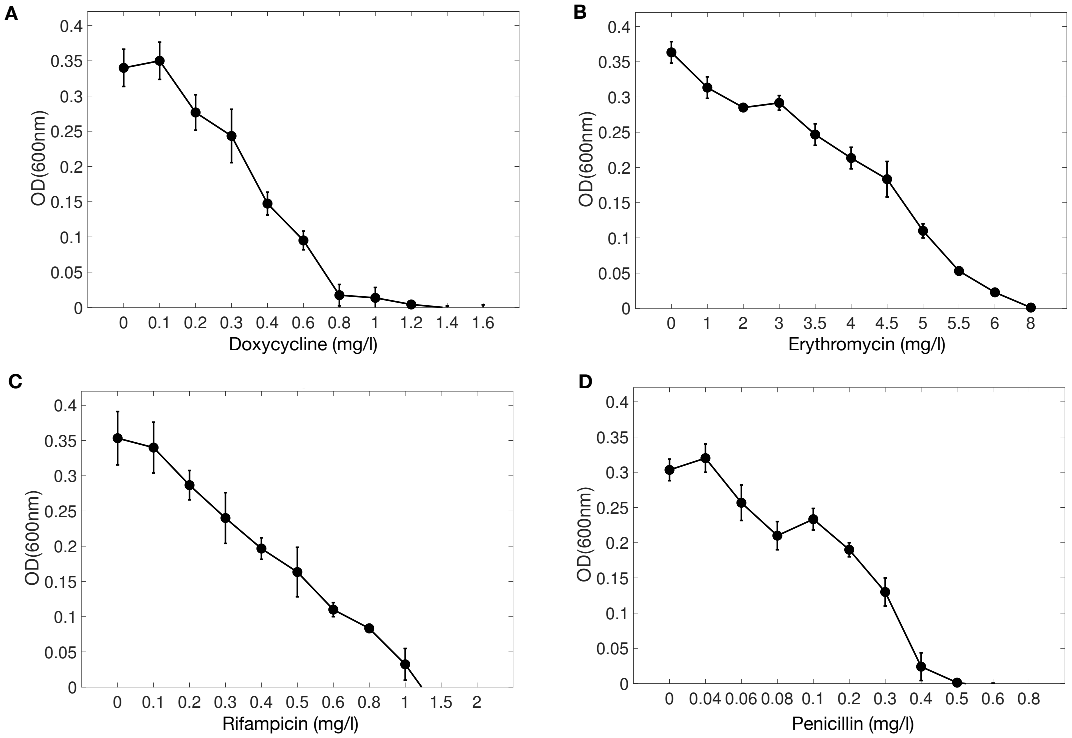

Fig. S1. Antibiotic dose responses for *E.coli*(MG1655) with a concentration gradient of (A) doxycycline, (B) erythromycin, (C) rifampicin and (D) penicillin (mean ± SE, *n* = *3*). OD was measured after a 24 hour incubation period (see Supplementary Methods).

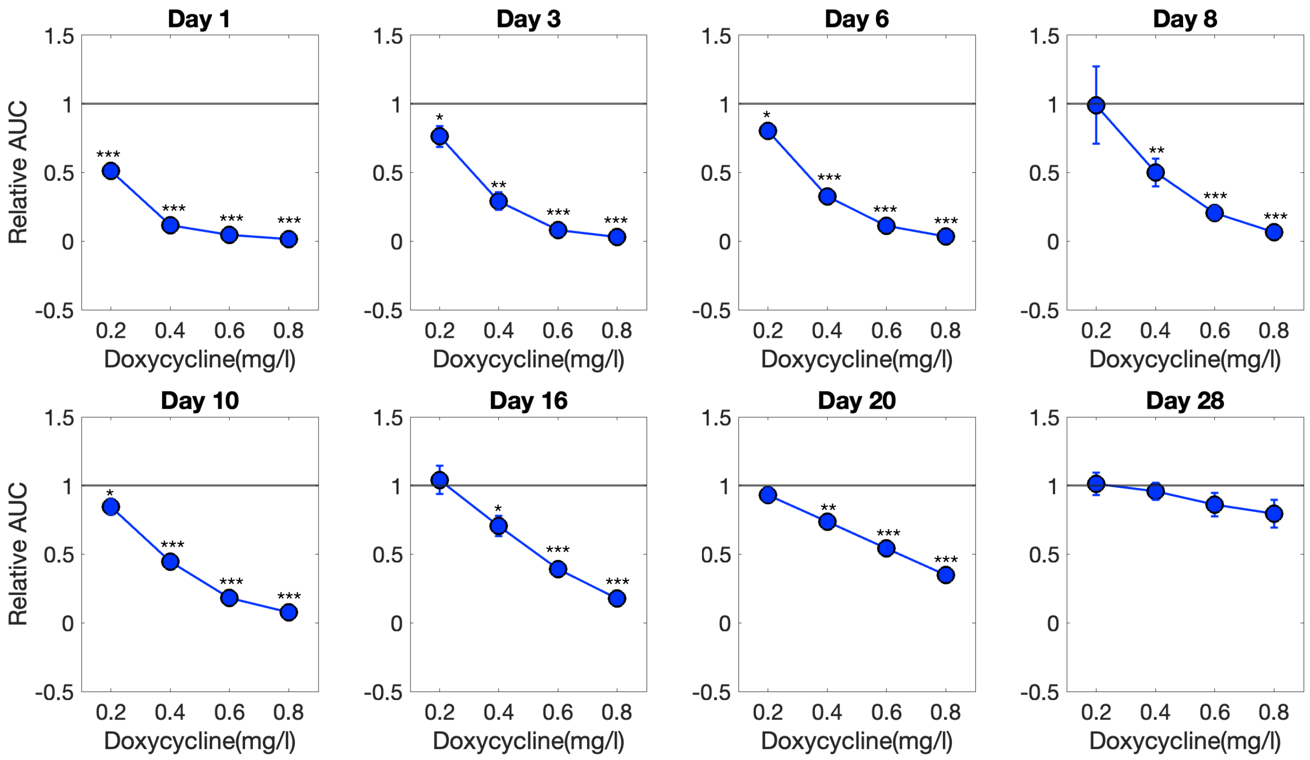

Fig. S2. The inhibitory activity of doxycycline decreases in media over 28 days, restoring the growth of *E.coli* (MG1655). During the long-term growth of *E.coli* in the TGD protocol (Figure 1), doxycycline was only used on the first day and so we asked how long doxycycline would retain its inhibitory properties in media for. Doxycycline exposures (at 0.2, 0.4, 0.6 and 0.8mg/L and a drug-free control) were prepared in M9 media and incubated at 30°C for 28 days. Periodically, 150 μL of media were removed and inoculated with MG1655, as well as 0.2% glucose and 0.1% casamino acids to test for growth inhibition by that media: when all those data lie on the line 'AUC = 1', the media is no longer inhibitory. Therefore, growth at all concentrations of doxycycline was all but restored by 28 days, as can be seen in the area under the growth curve data (AUC) which are shown for each drug concentration relative to drug free conditions (* = $p < 0.05$, ** = $p \leq 0.01$, *** = $p < 0.001$, one sample t-test, *n = 3*, mean $\pm$ SE).

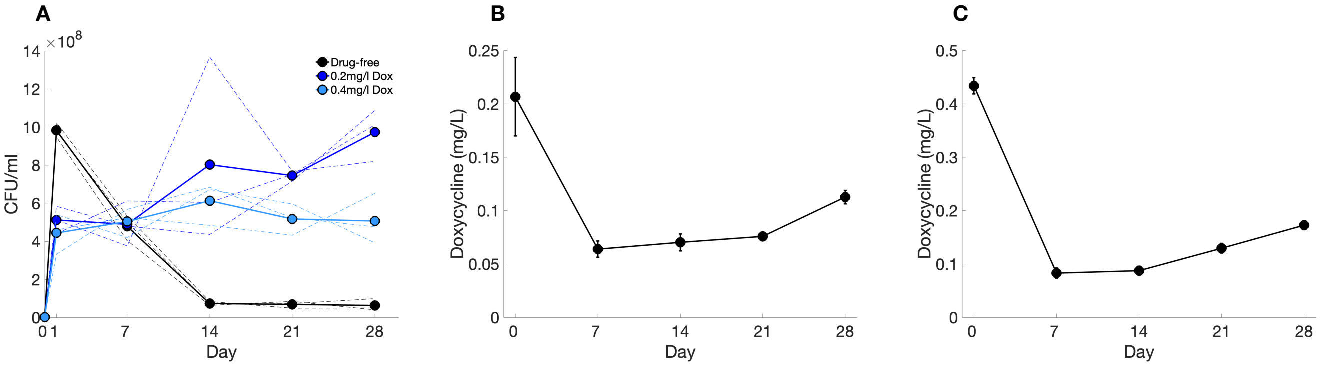

Fig. S3. The concentration of doxycycline in *E.coli* culture changes over 28 days. (A) Cultures of *E.coli* (MG1655) were initially exposed to doxycycline (0.2 or 0.4mg/L) or left-drug free, in triplicate. Cultures were incubated at 30°C for 28d without the addition of further antibiotic or nutrient. CFU/mL was then measured at regular intervals (days 0,1,7,14,21 and 28). The mean data is displayed as a solid line, with individual replicates shown as dashed lines, note how population densities are maintained at higher densities in the presence of the drug, consistent with the main text. LC-MS analysis was carried out at regular intervals (days 0,7,14,21 and 28) on the supernatant of cultures initially exposed to (B) 0.2 and (C) 0.4mg/L doxycycline (mean $\pm$ SE, $n = 3$). Extracellular doxycycline reduces in concentration by approximately 69% and 81% during week 1 in cultures initially exposed to 0.2 and 0.4mg/L doxycycline, respectively. The concentration then reaches a plateau at a lower value thereafter, achieving an approximate stasis, or even slight increase, for the remaining 3 weeks, reaching around 46% and 60% of the original concentration by day 28, respectively. Some evidence that extracellular doxycycline is increasing in concentration at later times is apparent which, we hypothesise, can be consistent with the release of intracellular drug from within dead and lysed cells.

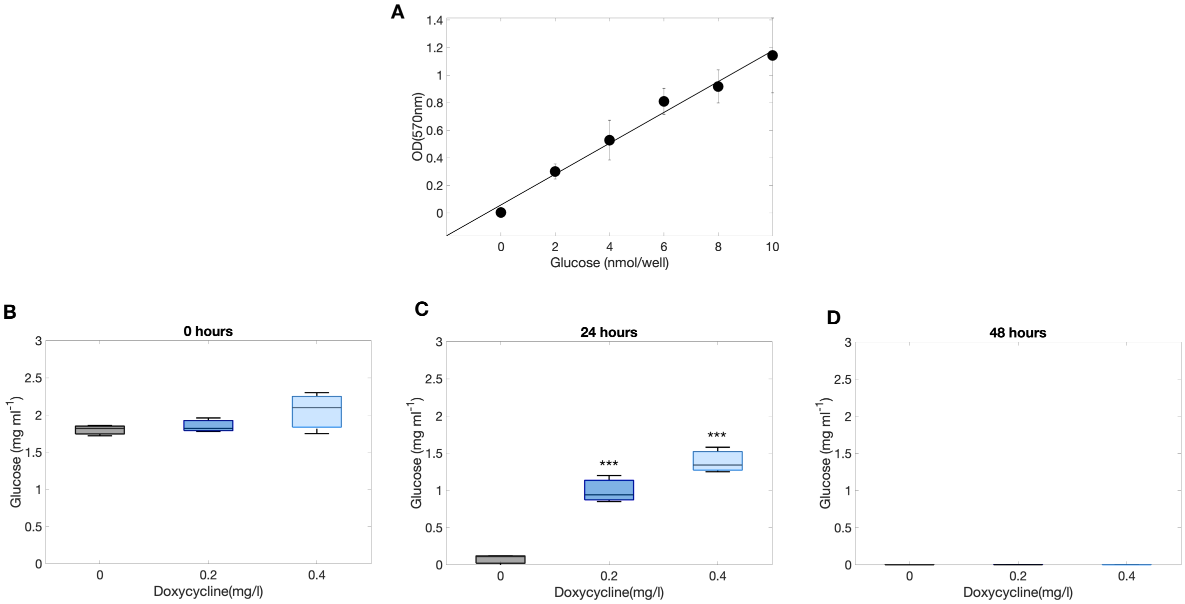

Fig. S4. Glucose is fully exhausted in all drug-free and doxycycline conditions by 48 hours. Supplementary Methods describes this assay. (A) A standard calibration curve was first determined for glucose concentration using a colorimetric glucose assay based on absorbance at 570nm measured at known glucose concentrations in triplicate. A linear regression was fitted to the data in order to infer future glucose concentrations. (B-D) The glucose concentration was determined from *E.coli* (MG1655) cultures exposed to 0, 0.2 and 0.4 mg/L of doxycycline for 72h. The same concentration of glucose was provided to all cultures at 0 hours (B) and by 24 hours (C) this has been depleted in drug-free conditions. By 48 hours (D) the remaining glucose in doxycycline-treated cultures has also been exhausted. The delay in glucose depletion within doxycycline-treated cultures is likely due to the growth inhibition. (2 sample t-test, *** = p <0.001, $n = 3$.)

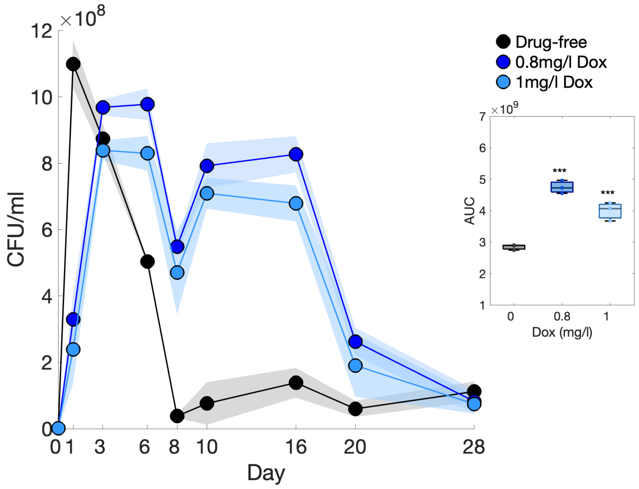

Fig. S5. Longevity benefits are observed in concentrations of doxycycline up to the IC$_{90}$ in *E.coli*(MG1655). Growth and decline data of MG1655 exposed to concentrations of doxycycline up to the IC$_{90}$ (0.8 and 1mg/L) or left drug-free for 28d without the addition of fresh nutrients or drug indicate that populations decay soonest when the drug is absent. Mean CFU/mL data is displayed as a solid line $\pm$ estimated 95% confidence intervals (CI) shown as a shaded area. (inset) Box plots show area under the curve of the above 3 datasets where the medians, first and third quartiles are shown (one way ANOVA: $F_{(2,6)}$ = 60.33, $p < 0.0001$, with post-hoc Tukey. Asterisks represent the p value of the post-hoc test which compares drug-free and doxycycline-treated populations: *** = $p < 0.001$, *n = 3*).

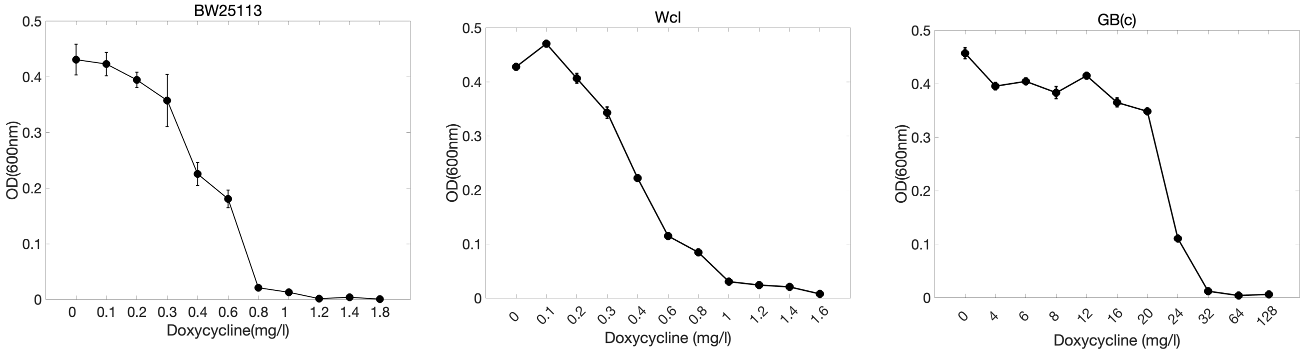

Fig. S6. Antibiotic dose responses for *E.coli* strains BW25113, Wcl and GB(c) with a concentration gradient of doxycycline (mean $\pm$ SE, $n = 3$). OD was measured after a 24 hour incubation period (see Supplementary Methods).

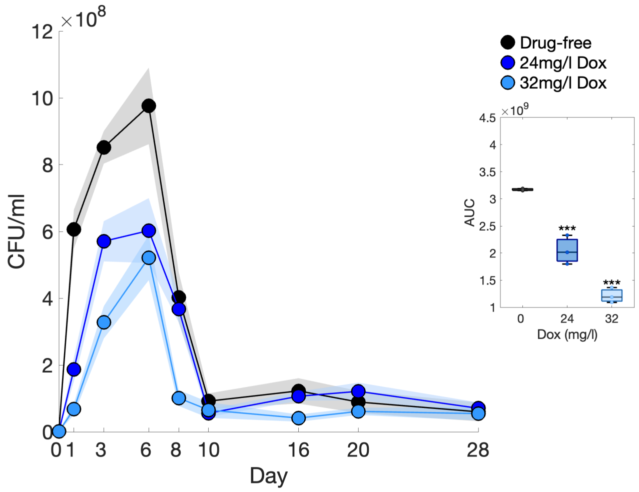

Fig. S7. Longevity benefits are absent in doxycycline-resistant *E.coli*(GB(c)) in concentrations of doxycycline up to the IC$_{90}$. Growth and decline data of strain GB(c) exposed to concentrations of doxycycline up to the IC$_{90}$ (24 and 32mg/L) or else left drug-free for 28d without the addition of fresh nutrients show the antibiotic reduces population density in an AUC measure. Mean AUC data are displayed as a solid line $\pm$ estimated 95% confidence intervals (CI) shown as a shaded area. The box plot insets show medians and first and third quartiles (one way ANOVA: $F_{(2,6)}$ = 95.88, $p < 0.0001$, with post-hoc Tukey. Asterisks represent the p value from the post-hoc test comparing drug-free and doxycycline-treated populations: *** = $p < 0.001$, *n* = *3*).

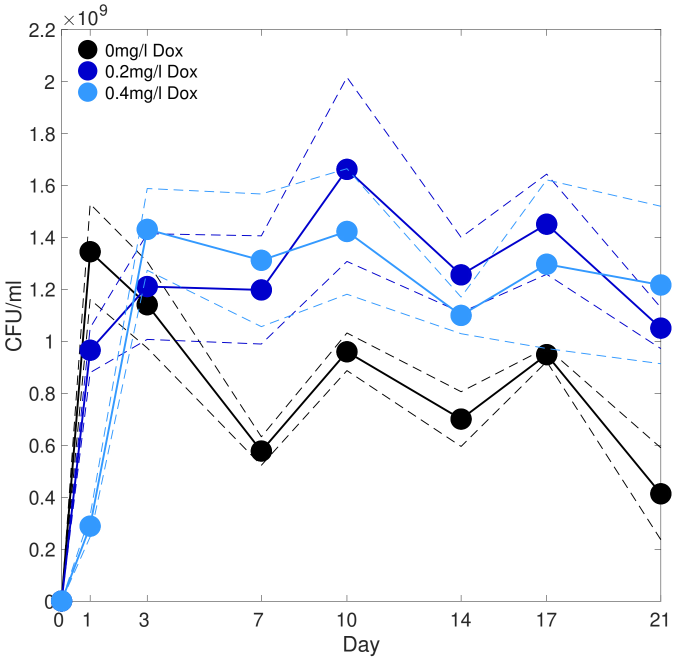

Fig. S8. The survival of *E.coli*(MG1655) over 21 days for the purpose of DNA sequencing. MG1655 was exposed to doxycycline (0.2 and 0.4mg/L) at inoculation or left drug-free for 21 days without the further addition of fresh nutrients or antibiotics. Samples were taken at regular intervals (days 1, 3, 7, 10, 15, 17 and 21) for the purposes of whole genome sequencing. Mean data are displayed as a solid line with individual replicates shown as dashed lines (*n=4*). Note that the growth and death dynamics within these populations differ from Figures 1 and S5. It is possible this is due to the larger volumes of media and culture vessel used to facilitate sequencing (500ml), indeed culture volume has been shown to impact bacterial growth and entry into death phase (1).

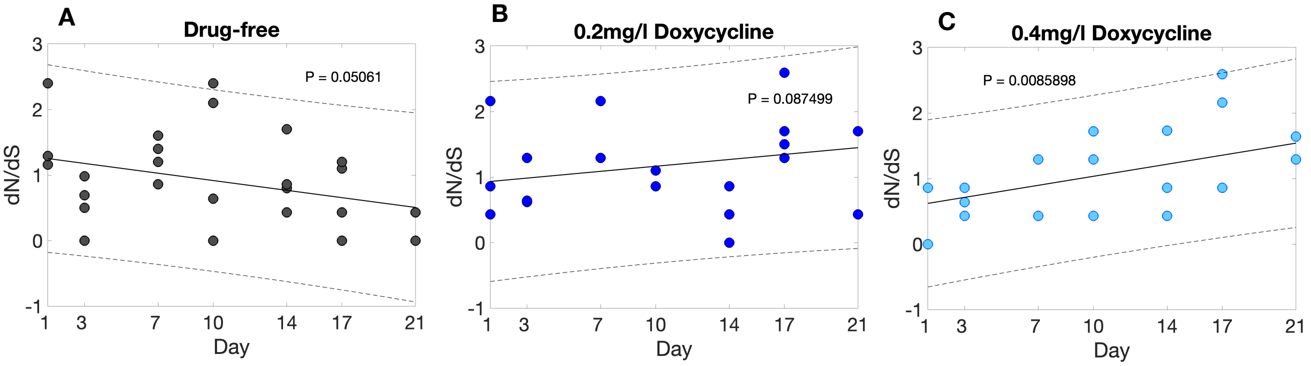

Fig. S9. dN/dS **ratios during 21 days of starvation in different doxycycline conditions based on cultures in Figure S8.** The trajectory of the dN/dS ratio was found to decrease in drug-free condition over 21 days, whilst it increased with both 0.2 and 0.4mg/L doxycycline, however this statistic is not significant. Note: a dN/dS value above 1 indicates an excess of nonsynonymous to synonymous mutations and therefore the mutations are likely to be adaptive. (Linear regressions are shown as a solid line $\pm$ estimated 95% confidence intervals shown as dashed lines, $n = 4$).

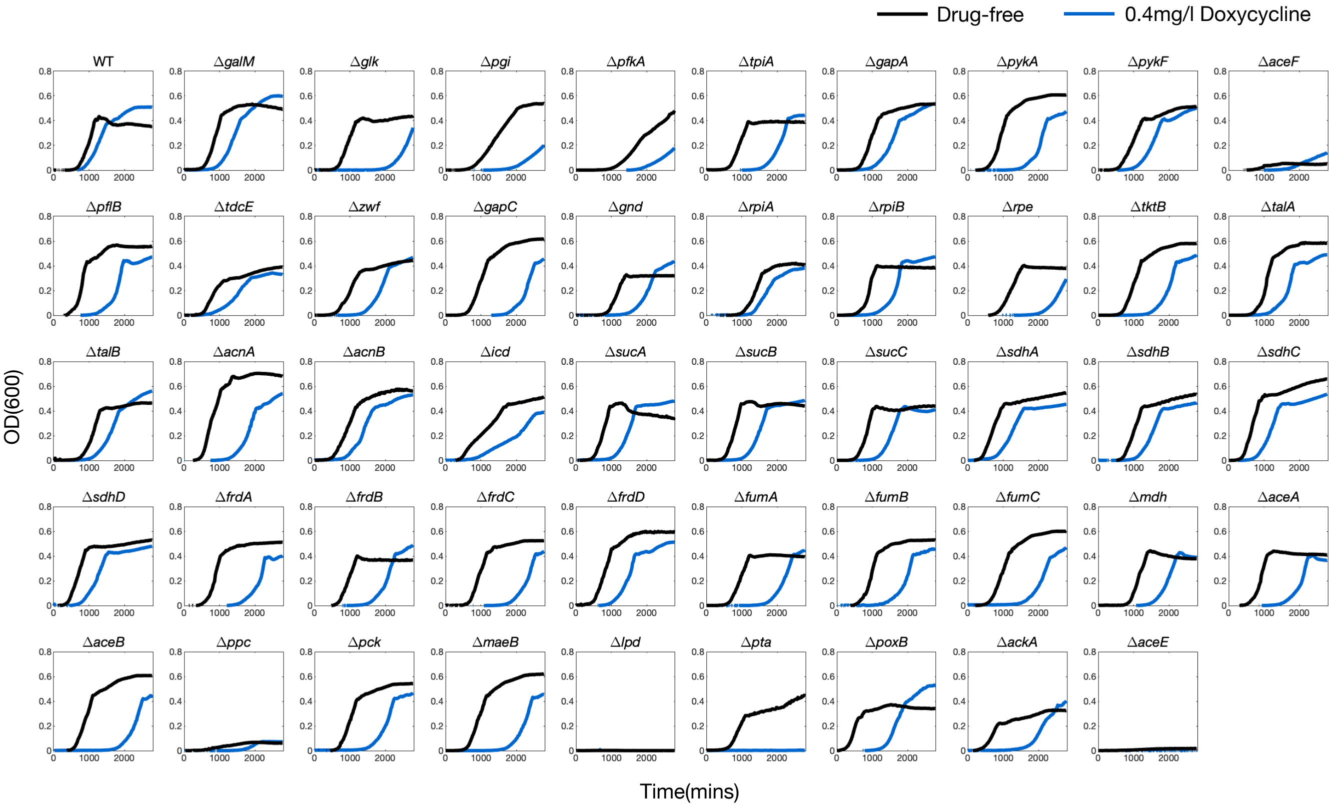

Fig. S10. Raw growth curves of metabolic gene knockout Keio strains in drug-free and doxycycline (0.4mg/L) conditions over 48 hours, used in Figure 5G.

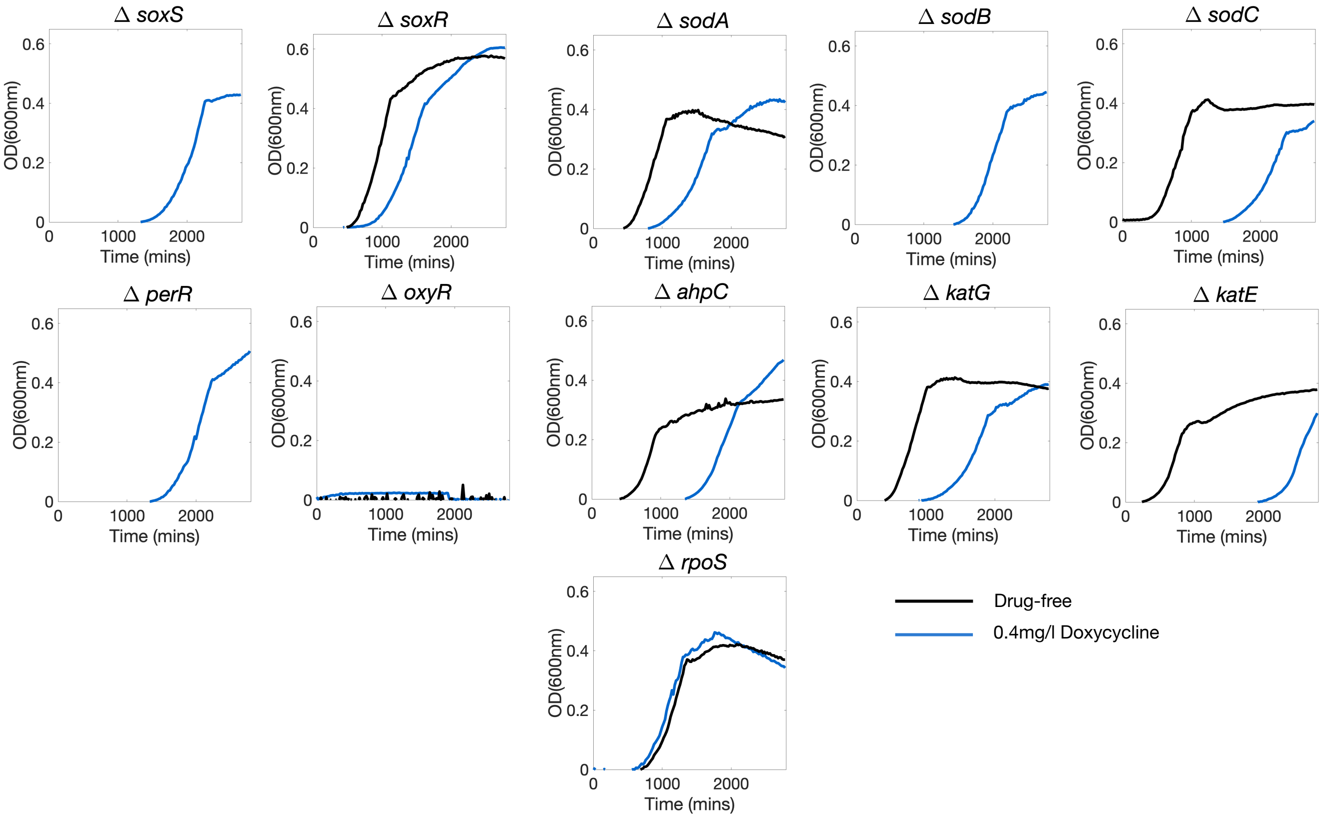

Fig. S11. Raw growth curves of ROS and stress-response gene knockout Keio strains in drug-free and doxycycline (0.4mg/L) conditions over 48 hours, used in Figure 5G.

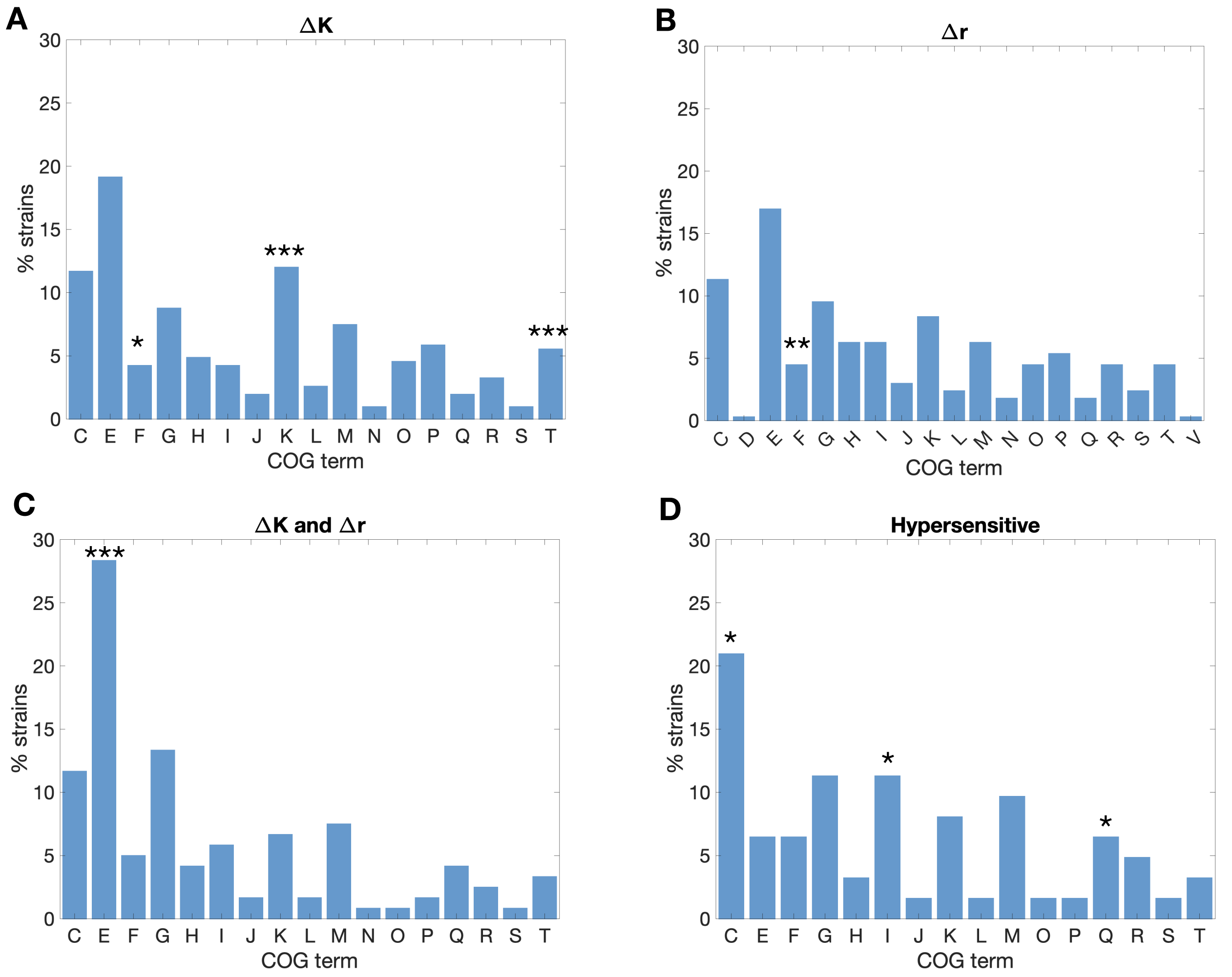

Fig. S12. Keio knockout strains with both phenotypic benefits and detriments in response to doxycycline are enriched in different COG terms. Doxycycline exhibits beneficial effects on some Keio knockout strains but it was detrimental to other strains (Figure 6). COG terms were assigned to gene and a Fishers exact test was used to determine if COG terms were enriched in any particular group of strains (note: * = $p < 0.05$, ** = $p < 0.01$, *** = $p < 0.001$). (A) 446 strains had benefits to ΔK relative to drug-free, i.e. $\Delta K > 1$, and these strains were found to be enriched in COG terms F $(p < 0.05)$, K $(p < 0.001)$ and T $(p < 0.001)$. (B) Δr was increased $(\Delta r > 1)$ relative to drug-free in 460 strains, enriched in COG term F $(p < 0.01)$. (C) 206 strains exhibit dual benefits to both ΔK and Δr and they were found to be enriched for COG term E $(p < 0.001)$. (D) 92 strains were hypersensitive to doxycycline in the sense that they only grew in the absence of the antibiotic. These hypersensitive strains were enriched for COG terms C $(p < 0.05)$, I $(p < 0.05)$ and Q $(p < 0.05)$. See Supplementary Table S5 for the COG terms and associated functions.

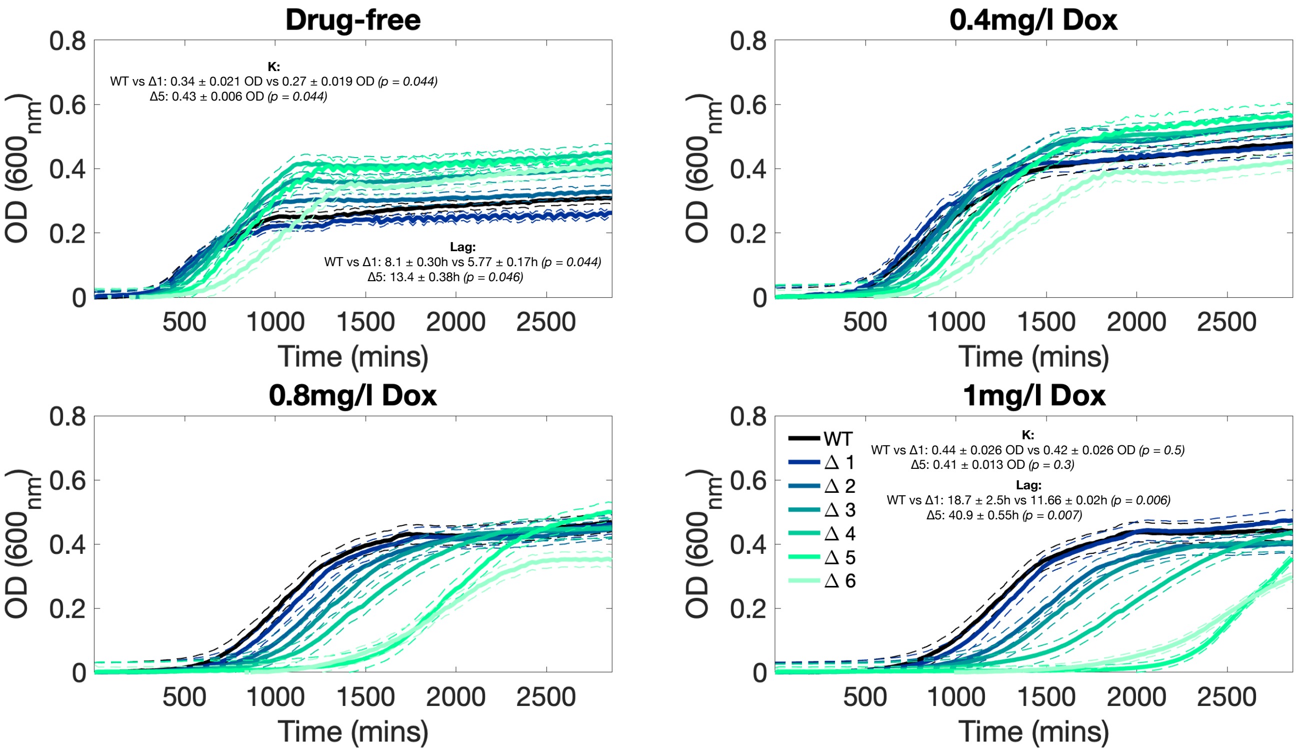

Fig. S13. Inhibition of *E.coli* by doxycycline is mediated by *rrn* copy number. Growth curves of the WT *E.coli*(MG1655) and *rrn* knockout strains in the presence and absence of doxycycline: in drug-free conditions, the removal of a single operon (6 x *rrn* operons, $\Delta 1$) is detrimental to K ($p < 0.009$), whilst those strains with fewer *rrn* operons, for example 2 x *rrn* operons ($\Delta 5$), have a longer lag period ($p < 0.046$), but a greater K ($p < 0.017$). With increasing concentrations of doxycycline, up to 1mg/L, the strains with fewer *rrn* operons (e.g. 2 x *rrn* operons, $\Delta 5$) lose the benefit to K but have a significantly longer lag phase than the WT ($p < 0.007$). A two-sided t-test was used to determine if there was a statistically significant difference in K and lag between the WT and *rrn* strains, with $p < 0.05$ indicating a significant difference. The bottom-right figure makes it clear that strains with more rrn operons are less sensitive to the antibiotic.

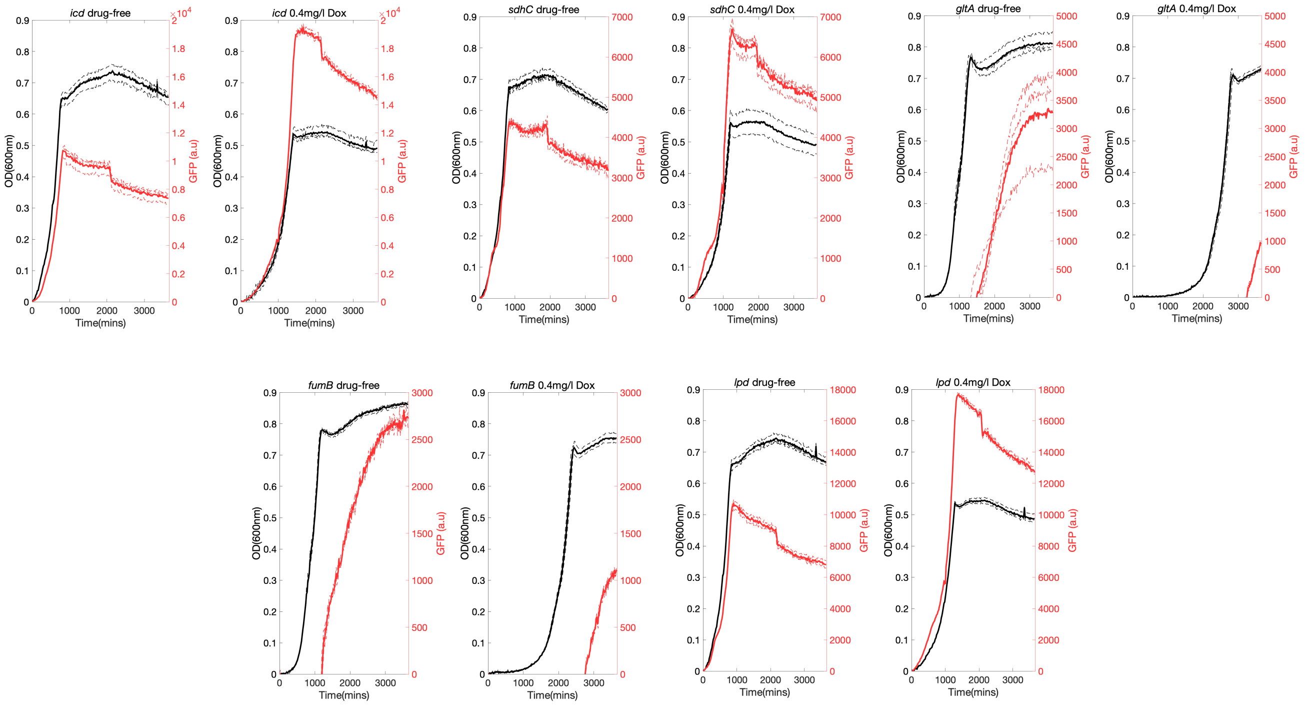

Fig. S14. Raw growth (OD) and GFP expression curves of *E.coli* TCA cycle GFP promoter strains in drug-free and doxycycline (0.4mg/L) conditions over 48 hours, as used in Figure 4A.

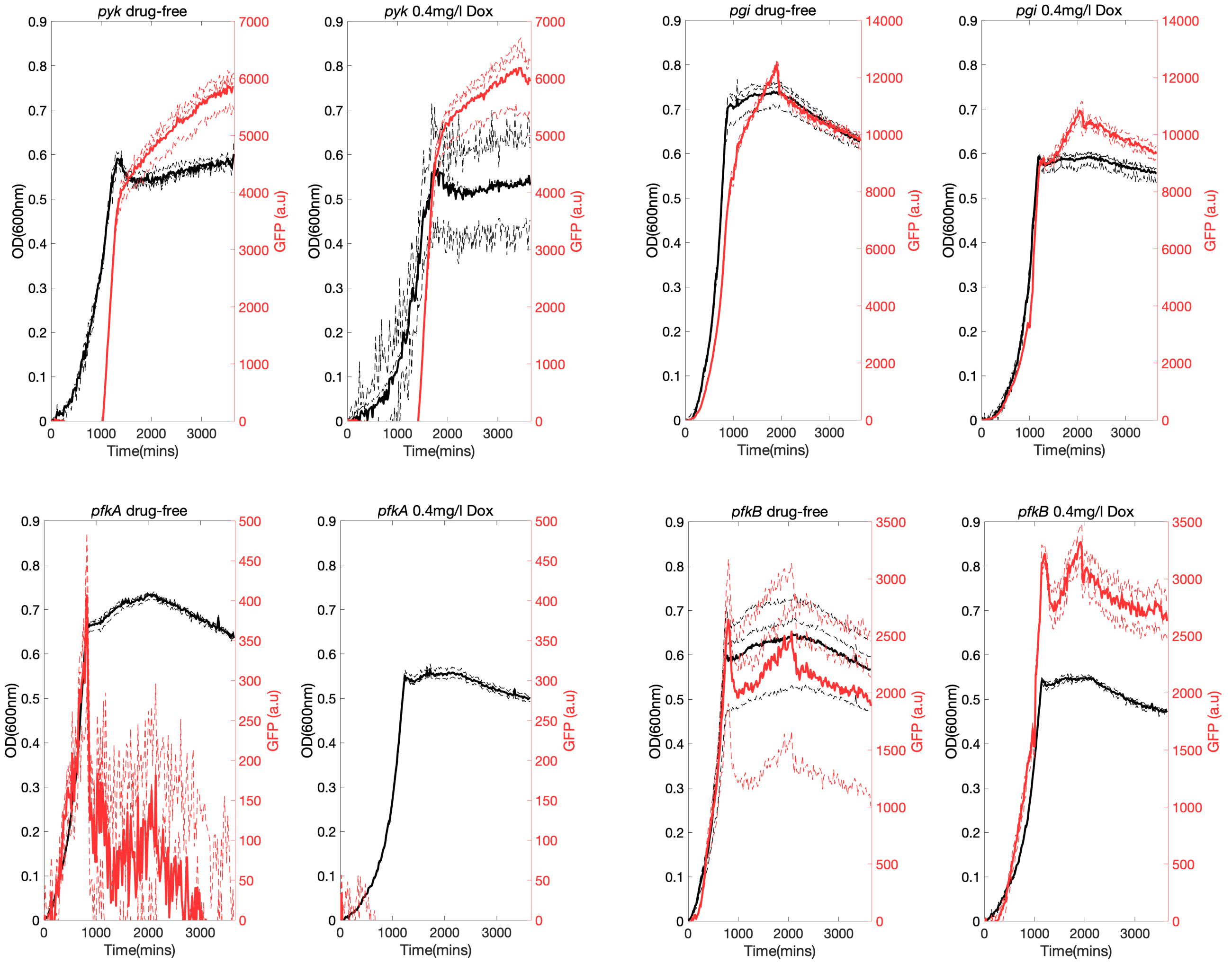

Fig. S15. Raw growth (OD) and GFP expression curves of *E.coli* glycolysis cycle GFP promoter strains in drug-free and doxycycline (0.4mg/L) conditions over 48 hours, as used in Figure 4B.

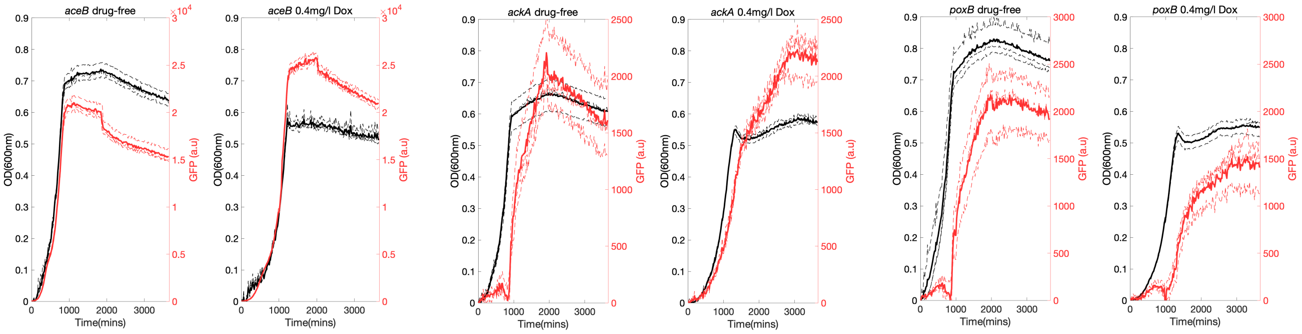

Fig. S16. **Raw growth (OD) and GFP expression curves of *E.coli* GFP promoter strains involved in acetate biosynthesis/metabolism in drug-free and doxycycline (0.4mg/L) conditions over 48 hours, as used in Figure 4C.**

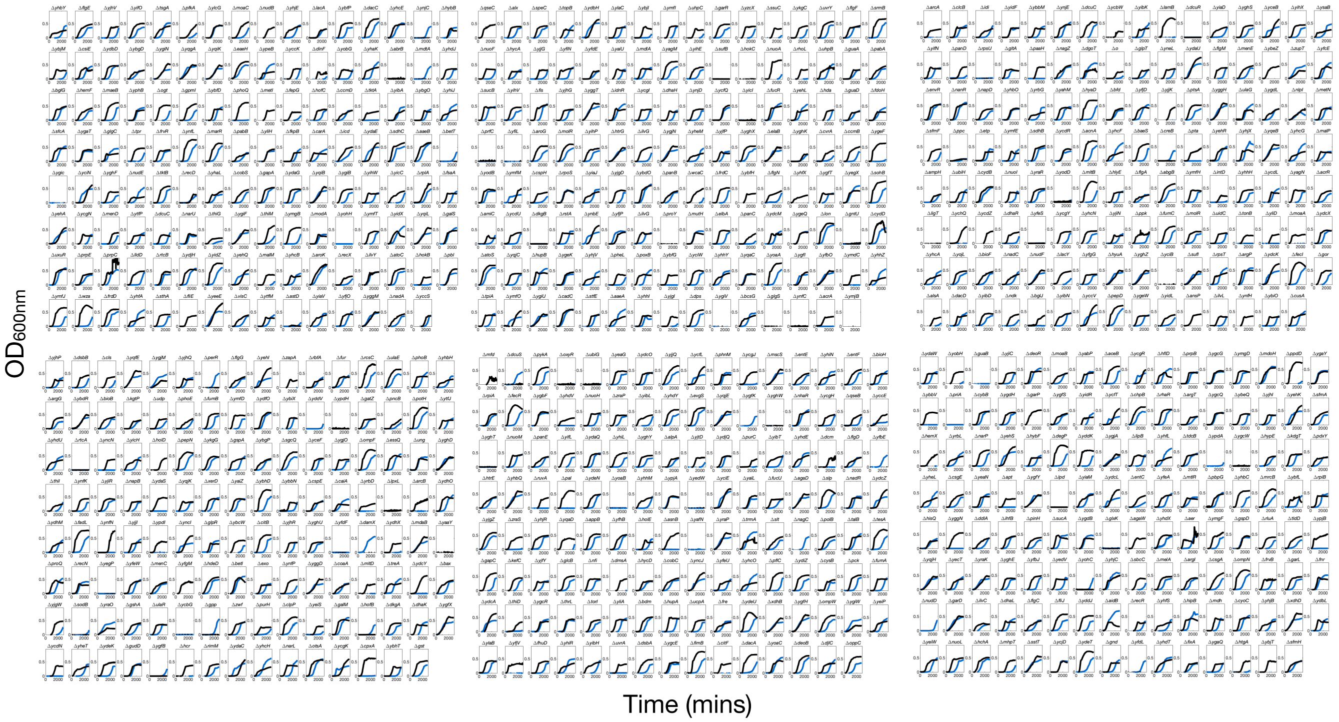

Fig. S17. Raw 48h growth curves of 768 Keio knockout strains in drug-free conditions and 0.4mg/L doxycycline, as used in Figure 6. Strains whose growth data indicate potential filamentation in either drug-free or doxycycline-treated conditions were removed from further analysis (Figure S19, see Supplementary Methods.)

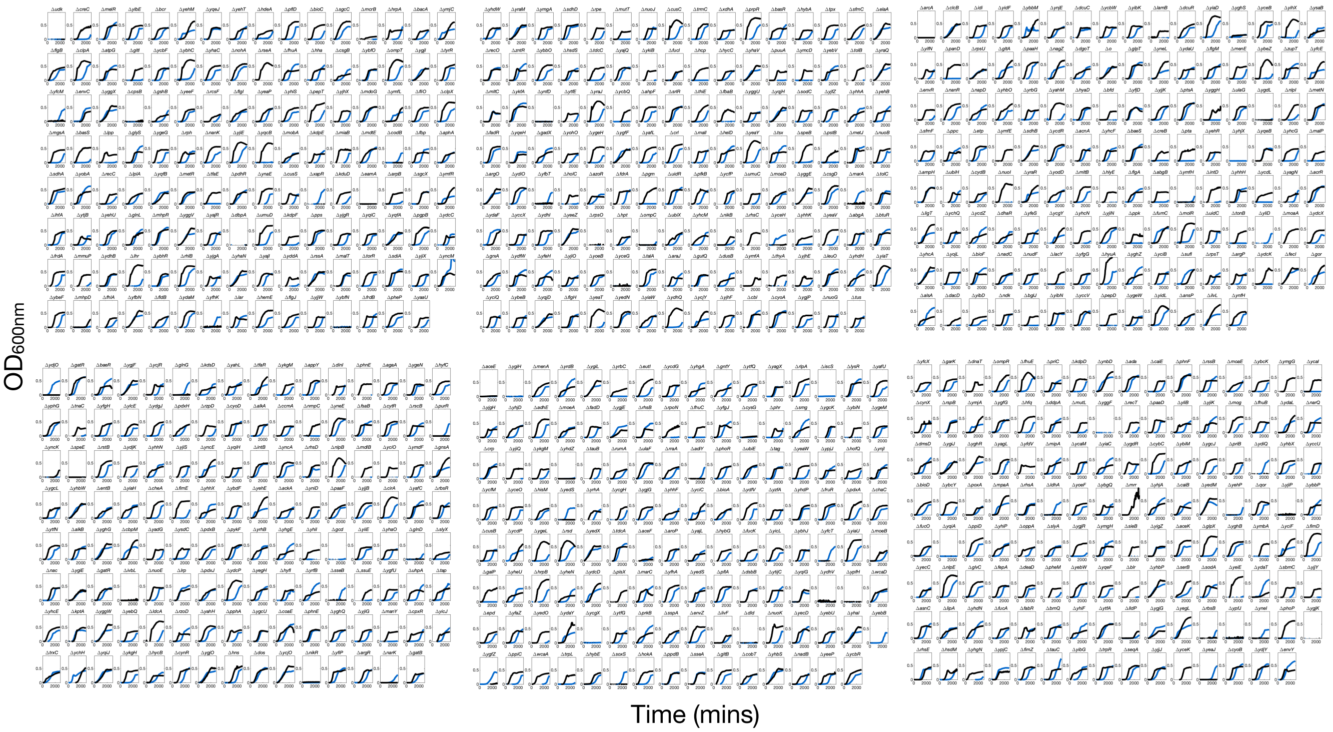

Fig. S18. **Raw 48h growth curves of 760 Keio knockout strains in drug-free conditions and 0.4mg/L doxycycline, as used in Figure 6**. Strains whose growth data indicate potential filamentation in either drug-free or doxycycline-treated conditions were removed from further analysis (Figure S19, see Supplementary Methods.)

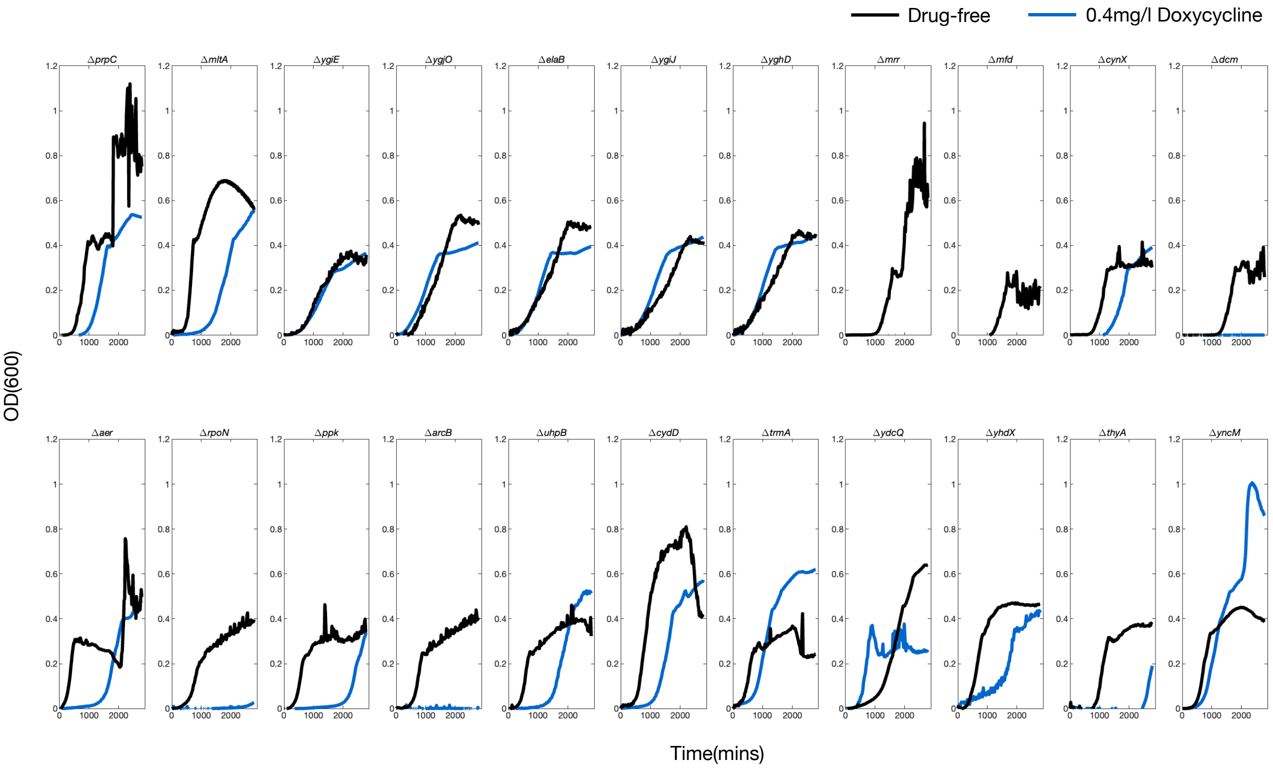

Fig. S19. Raw OD dynamics of 22 Keio strains are shown where an algorithm has detected suspected filamentation in either drug-free conditions or in treatments with 0.4mg/L doxycycline. The algorithm uses this approach: growth curves that deviate from an ideal growth kinetic were identified by taking the absolute difference between OD data and a logistic model (representing theoretically ideal population growth). The possible presence of filamentation was then inferred by taking the mean of the 'first difference' (akin to a derivative from the theory of calculus) of that absolute difference, as described in the Supplementary Methods where extensions of this algorithmic methodology are briefly discussed.

Day	Doxycycline(mg/l)	Replicate	Mean Coverage	Day	Doxycycline(mg/l)	Replicate	Mean Coverage
Ancestral	0	1	177.7	1	0.2	3	497.8
Ancestral	0	2	164.8	3	0.2	3	199.5
Ancestral	0	3	166.5	7	0.2	3	152.6
Ancestral	0	4	148.0	10	0.2	3	158.1
1	0	1	241.4	14	0.2	3	320.7
3	0	1	64.2	17	0.2	3	319.9
7	0	1	200.8	21	0.2	3	130.7
10	0	1	235.9	1	0.2	4	262.9
14	0	1	185.8	3	0.2	4	276.3
17	0	1	188.5	7	0.2	4	233.1
21	0	1	242.7	10	0.2	4	269.0
1	0	2	122.9	14	0.2	4	219.9
3	0	2	134.7	17	0.2	4	229.5
7	0	2	76.4	21	0.2	4	185.8
10	0	2	175.5	1	0.4	1	284.3
14	0	2	197.9	3	0.4	1	115.7
17	0	2	196.0	7	0.4	1	237.5
21	0	2	197.3	10	0.4	1	242.3
1	0	3	241.4	14	0.4	1	221.6
3	0	3	276.8	17	0.4	1	274.1
7	0	3	216.7	21	0.4	1	70.8
10	0	3	208.2	1	0.4	2	227.5
14	0	3	206.5	3	0.4	2	255.3
17	0	3	250.7	7	0.4	2	240.9
21	0	3	193.9	10	0.4	2	272.9
1	0	4	243.4	14	0.4	2	253.0
3	0	4	97.6	17	0.4	2	225.2
7	0	4	140.3	21	0.4	2	247.3
10	0	4	239.7	1	0.4	3	243.0
14	0	4	203.9	3	0.4	3	243.1
17	0	4	219.6	7	0.4	3	206.5
21	0	4	208.9	10	0.4	3	232.2
1	0.2	1	253.3	14	0.4	3	224.2
3	0.2	1	49.2	17	0.4	3	217.2
7	0.2	1	195.2	21	0.4	3	238.7
10	0.2	1	191.2	1	0.4	4	191.4
14	0.2	1	256.2	3	0.4	4	222.7
17	0.2	1	177.5	7	0.4	4	251.9
21	0.2	1	166.6	10	0.4	4	206.5
1	0.2	2	263.3	14	0.4	4	269.2
3	0.2	2	251.3	17	0.4	4	263.4
7	0.2	2	250.5	21	0.4	4	250.4
10	0.2	2	228.1				
14	0.2	2	206.3				
17	0.2	2	149.9				
21	0.2	2	225.8				

Table S1. These data are absolute mean coverages (i.e. the average (mean) depth of Illumina sequencing coverage across the entire genome as reported by the whole genome sequencing protocol (Methods F, Supplementary Methods 7.10)) for MG1655 genomes at all time points and concentrations of doxycycline tested (*n=4*).

Gene	Product	Doxycycline (mg/l)
rffG (L236L)	dTDP-glucose 4 6-dehydratase 2	0
eco/mqo (+295/+420)	Serine protease inhibitor ecotin/malate:quinone oxidoreductase	0 0.2
eco/mqo (+521/+194)	Serine protease inhibitor ecotin/malate:quinone oxidoreductase	0.2
nfuA (V130G)	Iron-sulfur cluster carrier protein NfuA	0 0.4
cyoB (G398V)	Cytochrome bo3 ubiquinol oxidase subunit 1	0
cyoB (M396V)	Cytochrome bo3 ubiquinol oxidase subunit 1	0.2 0.4
ftsE (R15S)	Cell division protein FtsE	0 0.2
ftsE (G20S)	Cell division protein FtsE	0.4
glgB (F156S)	1 4-alpha-glucan branching enzyme	0
adiY/adiA (-180/+145)	DNA-binding transcriptional activator AdiY/arginine decarboxylase	0.2
zntA/tusA (+47/+55)	Zn(2(+))/Cd(2(+))/Pb(2(+)) exporting P-type ATPase/sulfur transfer protein TusA	0.2
prpC (V337A)	2-methylcitrate synthase	0.2
murJ (V337A)	Putative lipid II flippase MurJ	0.2
msrP (P216P)	Periplasmic protein-L-methionine sulfoxide reductase catalytic subunit	0.2
dacD (I192V)	D-alanyl-D-alanine carboxypeptidase DacD	0.2
pstB/pstA (-56/+127)	Phosphate ABC transporter ATP binding subunit/phosphate ABC transporter membrane subunit PstA	0.2
ppc/argE (-349/+249)	Phosphoenolpyruvate carboxylase/acetylornithine deacetylase	0.2
yfaX (T222A)	Putative DNA-binding transcriptional regulator YfaX	0.2
rpoD (P531L)	RNA polymerase sigma 70 (sigma D) factor	0.2
glpT/glpA (-245/-28)	Sn-glycerol 3-phosphate:phosphate antiporter/anaerobic glycerol-3-phosphate dehydrogenase subunit A	0.2
glpT/glpA (-245/-28)	Sn-glycerol 3-phosphate:phosphate antiporter/anaerobic glycerol-3-phosphate dehydrogenase subunit A	0.4
insH-2 (S26N)	DLP12 prophage; IS5 transposase and trans-activator	0.2
cof/decR (+47/-106)	HMP-PP phosphatase/DNA-binding transcriptional activator DecR	0.2 0.4
gltP/yjcO (+361/+281)	Glutamate/aspartate : H(+) symporter GltP/Sel1 repeat-containing protein YjcO	0.4
ppc/argE (-518/+80)	Phosphoenolpyruvate carboxylase/acetylornithine deacetylase	0.2 0.4
lldD/trmL (+128/-70)	L-lactate dehydrogenase/tRNA (cytidine/uridine-2'-O)-ribose methyltransferase	0.4
ytcA (A80S)	Putative lipoprotein YtcA	0.4
guaB/xseA (-82/-80)	Inosine 5'-monophosphate dehydrogenase/exodeoxyribonuclease VII subunit XseA	0.4
recN (A50G)	DNA repair protein RecN	0.4
ybeL (H133H)	DUF1451 domain-containing protein YbeL	0.4
yhfZ/trpS (-248/+42)	Putative DNA-binding transcriptional regulator YhfZ/tryptophan–tRNA ligase	0.4

Table S2. SNPs identified in sequenced MG1655 populations at a frequency of 10%, or more, present in at least one time point and condition (0.2 or 0.4mg/L doxycycline, or drug-free) in the treatment-growth-death/starvation period of 21 days.

Gene	Product
icd	Isocitrate dehydrogenase
sdhC	Succinate dehydrogenase cytochrome b556 subunit
gltA	Citrate synthase
fumB	Fumarate hydratase
lpd	lipoamide dehydrogenase
pyk	Pyruvate kinase
pgi	Glucose-6-phosphate isomerase
pfkA	ATP-dependent 6-phosphofructokinase isozyme 1
pfkB	ATP-dependent 6-phosphofructokinase isozyme 2
aceB	Malate synthase A
ackA	Acetate kinase
poxB	Pyruvate dehydrogenase

Table S3. The *E.coli* promoter strains used in Figure 4.

Gene	Product
Δ*galM*	Aldose 1-epimerase
Δ*glk*	Glucokinase
Δ*pgi*	Glucose-6-phosphate isomerase
Δ*pfkA*	ATP-dependent 6-phosphofructokinase isozyme 1
Δ*tpiA*	Triosephosphate isomerase
Δ*gapA*	Glyceraldehyde-3-phosphate dehydrogenase A
Δ*pykF*	Pyruvate kinase I
Δ*aceF*	AceF-lipoate
Δ*pflB*	Formate acetyltransferase 1
Δ*tdcE*	Formate C-acetyltransferase
Δ*zwf*	Glucose-6-phosphate 1-dehydrogenase
Δ*gapC*	Glyceraldehyde-3-phosphate dehydrogenase
Δ*pykA*	Pyruvate kinase II
Δ*gnd*	6-phosphogluconate dehydrogenase
Δ*rpiA*	Ribose-5-phosphate isomerase A
Δ*rpiB*	Ribose-5-phosphate isomerase B
Δ*rpe*	Ribulose-phosphate 3-epimerase
Δ*tktB*	Transketolase 2
Δ*talA*	Transaldolase A
Δ*talB*	Transaldolase B
Δ*acnA*	Aconitate hydratase A
Δ*acnB*	Aconitate hydratase B
Δ*icd*	Isocitrate dehydrogenase
Δ*sucA*	2-oxoglutarate dehydrogenase E1 component
Δ*sucB*	dihydrolipoyltranssuccinylase
Δ*sucC*	Succinate-CoA ligase subunit beta
Δ*sdhA*	Succinate dehydrogenase flavoprotein subunit
Δ*sdhB*	Succinate dehydrogenase iron-sulfur subunit
Δ*sdhC*	Succinate dehydrogenase cytochrome b556 subunit
Δ*sdhD*	Succinate dehydrogenase hydrophobic membrane anchor subunit
Δ*frdA*	Fumarate reductase flavoprotein subunit
Δ*frdB*	Fumarate reductase iron-sulfur subunit
Δ*frdC*	Fumarate reductase subunit C
Δ*frdD*	Fumarate reductase subunit D
Δ*fumA*	Fumarate hydratase class I
Δ*fumB*	Fumarate hydratase class I
Δ*fumC*	Fumarate hydratase class II
Δ*mdh*	Malate dehydrogenase
Δ*aceA*	Isocitrate lyase
Δ*aceB*	Malate synthase A
Δ*ppc*	Phosphoenolpyruvate carboxylase
Δ*pck*	phosphoenolpyruvate carboxykinase
Δ*maeB*	NADP-dependent malic enzyme
Δ*lpd*	Dihydrolipoyl dehydrogenase
Δ*pta*	Phosphate acetyltransferase
Δ*poxB*	Pyruvate dehydrogenase
Δ*ackA*	Acetate kinase
Δ*aceE*	Pyruvate dehydrogenase E1 component
Δ*soxS*	DNA-binding transcriptional dual regulator SoxS
Δ*soxR*	Redox-sensitive transcriptional activator SoxR
Δ*sodA*	Superoxide dismutase
Δ*sodB*	Superoxide dismutase
Δ*sodC*	Superoxide dismutase
Δ*perR*	Peroxide Responsive Regulator
Δ*ahpC*	Alkyl hydroperoxide reductase C
Δ*katG*	Catalase-peroxidase
Δ*katE*	Catalase HPII
Δ*oxyR*	Hydrogen peroxide-inducible genes activator
Δ*rpoS*	RNA polymerase sigma factor RpoS

Table S4. The *E.coli* Keio knockout strains used in Figure 5G.

COG term	Function
A	RNA processing and modification
B	Chromatin Structure and dynamics
C	Energy production and conversion
D	Cell cycle control and mitosis
E	Amino Acid metabolism and transport
F	Nucleotide metabolism and transport
G	Carbohydrate metabolism and transport
H	Coenzyme metabolism
I	Lipid metabolism
J	Translation
K	Transcription
L	Replication and repair
M	Cell wall/membrane/envelope biogenesis
N	Cell motility
O	Post-translational modification.Protein turnover. Chaperone functions
P	Inorganic ion transport and metabolism
Q	Secondary Structure
T	Signal Transduction
U	Intracellular trafficking and secretion
Y	Nuclear structure
Z	Cytoskeleton
R	General Functional Prediction only
S	Function Unknown

Table S5. COG terms and associated functions (2).

SI Materials and Methods

Media and strains. Minimal media was prepared by combining 350g K_2HPO_4 and 100g KH_2HPO_4 in 1L DI water and 29.4g trisodium citrate, 50g $(NH_4)_2SO_4$ and 10.45g $MgSO_4$ in 1L DI water. These components were autoclaved and diluted accordingly into growth media. Media was supplemented with 0.2% glucose (w/v) and 0.1% casamino acids from filter sterilised 20% and 10% stock solutions.

Table S6 describes all of the strains that were used and the protocols that they are associated with.

Protocol	Strain	Genotype	Doxycycline IC90 (mg/l)	Ref
A C F	*E.coli* MG1655	K-12 *F- λ - ilvG0 rfb -50 rph -1*	0.8	(3)
B	*E.coli* GB(c)	MC4100 galK::CFP ampR pGW155B (Tetracycline resistance mechanism provided by pGW155B)	32	(4)
B	*E.coli* Wcl	MC4100 galK::CFP ampR pCS-λ (Kanamycin resistance mechanism provided by pCS lambda)	1.3	(4)
D	*E.coli* GFP promoter collection	MG1655. Various GFP promoter strains as detailed in Table S3	WT - 0.9	(5)
E	*E.coli* Keio collection	K-12 BW25113. Various knockout strains as detailed in Table S4 and Figures S17-S18	WT - 1.1	(6)
G	*E.coli* MG1655Δ 1 *rrn*	MG1655 Δ rrnE	-	(7)
G	*E.coli* MG1655 Δ 2 *rrn*	MG1655 Δ rrnGB	-	(7)
G	*E.coli* MG1655 Δ 3 *rrn*	MG1655 Δ rrnGBA	-	(7)
G	*E.coli* MG1655 Δ 4 *rrn*	MG1655 Δ rrnGBAD	-	(7)
G	*E.coli* MG1655 Δ 5 *rrn*	MG1655 Δ rrnGBADH ptRNA67	-	(7)
G	*E.coli* MG1655 Δ 6 *rrn*	MG1655 Δ rrnGBADHB ptRNA67	-	(7)

Table S6. The bacterial strains used throughout the paper.

CFU/OD calibration. Optical density (OD) has been used throughout as a proxy for cell number, however cell size can vary during different stages of growth and, given this, the robustness of OD measurements must be tested (8, 9). A relationship between OD and CFU/ml was therefore examined for *E.coli*(MG1655) to ensure that there is a linear relationship between these two measurements in our culture conditions.

Accordingly, 24h cultures of *E.coli*(MG1655) were implemented in defined minimal media after which 6 serial dilutions were performed and OD(600nm) reads were taken using a Tecan Spark microplate reader. To quantify CFU/ml, 10µl of diluted culture (with final dilutions of 10^{-5} and 10^{-6}) were spread onto LB agar plates and incubated at 30°C for 24h. The number of colonies were then counted and the following calculation was used to determine the number of cells in the original culture:

$$\text{CFU/ml} = \frac{\text{Colony number} \times \text{Dilution factor}}{\text{Volume of culture plated (10 µl)}}. \qquad [1]$$

The resulting CFU-to-OD calibration curve is shown in Figure S20.

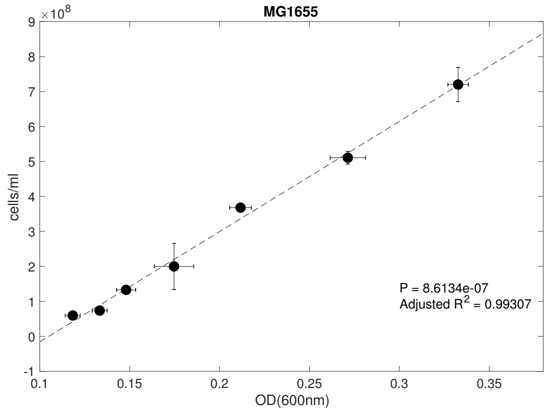

Fig. S20. A calibration curve for comparisons of OD and CFU/mL determined for *E.coli*(MG1655), dashed line is linear regression (mean ± SE, *n = 3*). This indicates a high degree of correlation between CFU/mL and OD where population densities are high. However, because the dashed line does not pass through the origin $(0, 0)$, it appears OD may over-estimate population densities in the regime where CFU/mL measurements are low.

Measurement of growth parameters. Throughout, growth rate (r) and carrying capacity (K) measurements were estimated using the logistic equation, as described previously (10):

$$\frac{dN}{dt} = rN\left(1 - \frac{N}{K}\right) \qquad [2]$$

which has also been modified to account for the lag phase; here $\mathrm{d}N/\mathrm{d}t$ is the microbial per capita growth rate. To use this in practise, growth rate and carrying capacity parameters (the maximum possible population density realised by the logistic

model) were determined from the best fit of a four-parameter model against optical density (OD) population growth data:

$$N(t) = B + \frac{K}{1 + a \cdot e^{-r(t-L)}} \quad [3]$$

Here $N(t)$ is the cell density at time t, a represents biological features that are not important for arguments in the main text, L is lag, B is blank parameter used to fit $N(t)$ to bacterial density data and this, note, includes the blank component due to the non-zero, non-biological contribution plastic microtitre plates make to OD measurements.

Dose response. The following antibiotics were used in this study: doxycycline (Sigma, United States), erythromycin (Duchefa Biochemie, Netherlands), penicillin (Sigma, United States) and rifampicin (Melford, United Kingdom). The solvent and stock solution concentrations were prepared for each according to the manufacturers instructions, as displayed in Table S7. All antibiotic stock solutions were then filter sterilised using a 0.22 µm filter before being frozen at -20°C. Working solutions were prepared from stock solutions by dilution in M9 or M9CAA. Stock solutions were stored for no longer than 2 weeks before use and each aliquot was only used once. Working solutions were prepared on the day of use and not stored long-term.

Antibiotic	Solvent	Stock solution	Storage temperature
Doxycycline	DI water	5mg/ml	-20°C
Erythromycin	Pure ethanol	10mg/ml	-20°C
Rifampicin	Pure methanol	25mg/ml	-20°C
Penicillin	DI water	10mg/ml	-20°C

Table S7. Antibiotic stock solutions.

Dose response curves were produced to determine IC_x values for each antibiotic and to estimate an MIC. Linear gradients of doxycycline, erythromycin, rifampicin and penicillin were first prepared in microtiter plates and diluted 1:1 in M9CAA inoculated with 1x10^6 CFU/ml of the appropriate *E.coli* strain. The microplates were then incubated at 30°C, shaken at 160rpm and OD(600nm) readings were taken every 20 minutes in a Tecan Spark microplate reader for 24h.

Inhibitory activity of doxycycline over 28 days. *E.coli*(MG1655) was cultured in long-term, 28d growth experiments (TGD protocols) using media that was initially supplemented with antibiotic (Figure 1A). Given the lengthy duration of these protocols we estimated the time until the complete loss of doxycycline's inhibitory activity. For this, 15ml M9 media was supplemented with doxycycline (0.2, 0.4, 0.6 or 0.8 mg/L) or left drug-free and incubated at 30°C in a shaking incubator (160rpm). At set time points (days 1, 3, 6, 8, 10, 16, 20 and 28), 150μl was removed (in triplicate) for each doxycycline concentration and inoculated with 1x10^6 CFU/ml of fresh overnight MG1655 culture, as well as 0.2% glucose and 0.1% casamino acids, into a 96-well microplate. These cultures were grown for 24hrs in a Tecan Spark microplate reader at 30°C and growth was measured as OD(600nm). The degree of inhibition of this sample was then calculated by determining AUC of the resulting growth data in doxycycline exposures and dividing these by the AUC of growth kinetic data observed in drug-free conditions. When these result in a value of unity, growth inhibition has ceased.

Note that doxycycline's inhibitory activity here could be effected by the presence of *E.coli* during long-term growth TGD experiments however, here, it would not be feasible to include *E.coli* during the incubation period, as although the cells could be removed to allow for a fresh dose response to be performed, the final amount of glucose and casamino acids would be unclear.

Measurement of doxycycline concentration over 28 days in *E.coli* culture. The concentration of doxycycline was measured in *E.coli* TGD protocols lasting 28d by applying liquid chromatography mass spectrometry (LC-MS). *E.coli* (MG1655) (1x10^6 cells/ml) was first inoculated into 30ml of M9CAA in triplicate, supplemented with doxycycline (0.2 or 0.4mg/L) or left drug-free. The cultures were incubated at 30°C and shaken at 160pm for 28d, without the addition of further nutrients or doxycycline. At regular intervals (days 0,1,7,14,21 and 28), 10 μL was removed, serially diluted and spread onto LB agar plates for the purpose of colony counting. On days 0,7,14,21 and 28 an additional 2mL was removed from cultures that were initially supplemented with 0.2 or 0.4mg/L doxycycline for LC-MS analysis. These 2mL samples of culture were centrifuged at 4,300×g for 5 minutes and the supernatant was frozen at -20°C prior to LC-MS analysis. A calibration curve to determine the drug concentration was produced using a range of 13 known doxycycline concentrations (based on the range 0.4-0.004mg/L).

LC-MS Analysis. LC-MS analysis was undertaken using an Agilent 6410B triple quadrupole (QQQ) mass spectrometer coupled to a 1200 series HPLC (LC) system (Agilent Technologies, Santa Clara, USA). The LC conditions were as follows: chromatographic analyte separation was undertaken by loading 10 μL of sample onto an Agilent Poroshell 120 EC-C18, 2.7 μm, 30 x 150mm HPLC column. LC mobile phase A was 70 % water and mobile phase B was 30% acetonitrile, both were modified with 0.1% acetic acid and 0.1%, ammonium acetate (all solvents and modifiers were LC-MS grade). A gradient was initiated at time zero as follows: 0 min - 30% B; 9 min - 70% B; 12 min- 99% B; 18 min - 30% B; followed by 2 min re-equilibration time, at a flow rate of 0.3 mL min^{-1} and with the HPLC column maintained at 25°C for the duration. The QQQ mass spectrometer was

operated in positive ion mode using electrospray ionisation (ESI+). The gas temperature was 350°C, drying gas flow rate was 11L min^{-1}, nebuliser pressure was 35 psi and capillary voltage 4 kV.

MS Data analysis. MS data was acquired by Agilent MassHunter Acquisition software (version B.08.00) operated in Multiple Reaction Monitoring (MRM) mode and analysis was undertaken using Agilent MassHunter Quantitative Analysis software (version B.07.01, SP1).

Glucose availability assay. To assess whether all available glucose was exhausted from media, for instance in the TGD protocol, a colourmetric glucose assay (BioVision) was used. Briefly, a standard curve was produced using predetermined concentrations of glucose. 50μL of *E.coli*(MG1655) grown in either drug-free MC9AA or supplemented with 0.2 or 0.4 mg/L doxycycline (*n=3*) was extracted from culture at 0, 24 and 48h. Samples were loaded into a 96-well microplate with glucose assay master mix and incubated at 37°C for 30mins. Absorbance was read at 570nm in a Tecan Spark plate reader. The readings were then compared against the standard curve to determine the concentration of glucose in the sample and the following calculation was used to determine nmol/μL, where B is the amount of glucose in the sample well (nmol), V is the volume of sample in the well and D is the sample dilution factor used:

$$\text{Glucose concentration (nmol/ µl)} = \frac{B}{V \times D}. \quad [4]$$

Reactive oxygen species (ROS) assay. The ROS-sensitive dye *5(6)*-carboxy-2′,7′-dichlorodihydrofluorescein diacetate (Carboxy-H_2DCFDA, ThermoFisher Scientific, 10 μm) was used to measure the levels of intracellular ROS within *E.coli*(MG1655) cultures in 10ml M9CAA using a spectrophotometer, supplemented with doxycycline (0.2 and 0.4mg/L) or left drug-free (*n=12*). Fluorescence intensity (a proxy for ROS concentration) was measured after 24h of growth at 30°C, shaken at 160rpm. Three drug-free cultures lacking the fluorescent dye were used as a control for autofluorescence. Fluorescence was measured using a Tecan Infinite 200 Pro microplate reader (Ex/Em : 494/520nm).

Identification of Keio strains exhibiting filamentation. Some Keio strains might exhibit filamentation under antibiotic treatment, for example where gene knockouts are involved in cell division. This is relevant to our chosen method of growth measurement, OD, as filamentation is likely to impair the accuracy of OD data as a proxy for population size where OD changes are the result of filamentation, not an increase in cell number.

To mitigate this, we note that filaments of different lengths can result in large amounts of noise within OD data and, to detect this, we assessed growth curves based on OD data in the following way. First we define the functional known mathematically as the Sobolev p-norm of a datastream (i.e. of the function, $f(t)$) defined for $t \in [0, T]$ hours, which is a continuous time variable satisfying $f(0) = 0$:

$$\|f\|_{W_0^{1,p}} = \left(\int_0^T \left| \frac{df}{dt} \right|^p dt \right)^{1/p}.$$

Now let $d(t)$ be one datastream representing OD data and let $\ell(t)$ be an idealised, noise-free model of that data, which we choose to be a logistic function (3) fitted to that data. We assume $d(0) = \ell(0)$ is satisfied so that the logistic and OD data have the same initial density when $t = 0$.

OD datastreams with filamentation are likely to deviate far from an ideal, noise-free logistic and so $\|d-\ell\|_{W_0^{1,p}}$ is a candidate metric for detecting filamentation. As OD data are discrete, not continuous, we therefore re-formulate $\|d-\ell\|_{W_0^{1,p}}$ as the sum of the absolute difference between OD data and the idealised logistic model, something that can be written in Matlab notation as

$$F(d) := (\texttt{sum}(\texttt{abs}(\texttt{diff}(d - \ell).\wedge\, p)))\ .\wedge\ (1./p) \quad [5]$$

which defines our filamentation-detection metric.

This definition ignores (i.e. scales away) the constant parameters T and the rate at which OD data are measured and the theoretical model, $\ell(t)$, is evaluated here at the same timepoints as d (i.e. when the OD data have been sampled). The value p can be changed to prioritise different deviations of the data from the ideal model and we chose $p = 1$ to give small and large deviations equal weight. Thereafter, OD data from strains whereby $F(d) > 1$ in either drug-free or doxycycline conditions were visually inspected for filamentation and, subsequently, all were excluded from further analysis.

Whole genome sequencing of *E.coli*(MG1655) cultures. *E.coli*(MG1655) were assayed in a TGD protocol for 21d (*n=4*) in drug free conditions and after exposure to doxycycline (0.2 or 0.4mg/L) without the later addition of nutrients or antibiotics. Larger culture volumes (500ml) than those used in Methods A were required to allow for the removal of culture samples for sequencing. At 7 time points over the course of the 21 days (Days 1, 3, 7, 10, 15, 17 and 21), 5ml of culture was removed and 10μl was spread on M9CAA agar plates for colony counting. The remaining culture was centrifuged at 2400×g, the supernatant was removed and the bacterial pellet was stored at -80°C in preparation for DNA extraction. Four replicates of the ancestral strain (*E.coli*(MG1655)) were also prepared and stored as cell pellets at -80°C in preparation for sequencing to ensure conformity between the published *E.coli*(MG1655) reference and the strain used here. In addition, CFU counts were measured for cultures in drug-free conditions that did not have any volume removed for the purposes of sequencing, this served as a control for the impact of volume loss on population dynamics.

DNA extraction. A GeneJet DNA purification kit (ThermoFisher) was used to extract DNA from the frozen bacterial pellets using a silica-based spin column method. Alterations to the standard protocol included an extended incubation time at 56°C to allow for complete cell lysis and an additional final elution step to maximise the DNA yield. The DNA was run on a 1% agarose gel to check that the DNA was not heavily fragmented and to check for protein contamination. The DNA was then quantified using the qubit system with the Qubit high sensitivity (HS) assay kit (ThermoFisher). The samples were then spun on a vacuum centrifuge (SpeedVac) at 50°C and pellets resuspended in 150μL elution buffer. The DNA was then stored at 4°C in preparation for sequencing. Paired-end libraries were prepared at the Institute of Clinical Molecular Biology (IKMB), Kiel University and run on the HiSeq using the Nextra library preparation protocol.

DNA quality control, mapping and variant calling. FastQC (v 0.11) was used to assess multiple quality control parameters such as read quality and GC content. Low quality reads (with a read quality below 20) were trimmed using Cutadapt (v2.10) (11). The reads were then mapped against the previously published *E.coli*(MG1655) reference genome using burrows-wheeler aligner (BWA, v0.7.4) (12) with standard parameters. The alignments were then sorted into genomic position and indexed using Samtools (v1.3.1).

Breseq v(0.30.0) (13) was used in polymorphism mode to call variants such as single nucleotide polymorphisms (SNPs). Varscan (v2.4.0) (14) was also used with a minimum average quality of 30. The annotation files for *E.coli*(MG1655) were accessed from NCBI and these Genbank files were used to annotate the polymorphisms identified. A combination of bespoke MATLAB and Python scripts were used for downstream analyses.

References

1. KE Kram, SE Finkel, Culture volume and vessel affect long-term survival, mutation frequency, and oxidative stress of *Escherichia coli. Appl. Environ. Microbiol.* **80**, 1732–1738 (2014).
2. RL Tatusov, MY Galperin, DA Natale, EV Koonin, The COG database: a tool for genome-scale analysis of protein functions and evolution. *Nucleic Acids Res.* **28**, 33–36 (2000).
3. FR Blattner, et al., The complete genome sequence of *Escherichia coli* k-12. *Science* **277**, 1453–1462 (1997).
4. R Chait, S Shrestha, AK Shah, JB Michel, R Kishony, A differential drug screen for compounds that select against antibiotic resistance. *PLOS ONE* **5**, 1–8 (2010).
5. A Zaslaver, et al., A comprehensive library of fluorescent transcriptional reporters for *Escherichia coli. Nat. Methods* **3**, 623–628 (2006).
6. T Baba, et al., Construction of *Escherichia coli* k-12 in-frame, single-gene knockout mutants: the keio collection. *Mol. Syst. Biol.* **2**, 2006.0008 (2006).
7. T Bollenbach, S Quan, R Chait, R Kishony, Nonoptimal microbial response to antibiotics underlies suppressive drug interactions. *Cell* **139**, 707–718 (2009).
8. H Makinoshima, et al., Growth phase-coupled alterations in cell structure and function of *Escherichia coli. J. Bacteriol.* **185**, 1338–1345 (2003).
9. T Nyström, Stationary-phase physiology. *Annu. Rev. Microbiol.* **58**, 161–181 (2004) PMID: 15487934.
10. C Reding-Roman, et al., The unconstrained evolution of fast and efficient antibiotic-resistant bacterial genomes. *Nat. Ecol. & Evol.* **1**, 0050 (2017).
11. M Martin, Cutadapt removes adapter sequences from high-throughput sequencing reads. *EMBnet.journal* **17**, 10–12 (2011).
12. H Li, R Durbin, Fast and accurate short read alignment with Burrows–Wheeler transform. *Bioinformatics* **25**, 1754–1760 (2009).
13. DE Deatherage, JE Barrick, *Identification of Mutations in Laboratory-Evolved Microbes from Next-Generation Sequencing Data Using breseq.* (Springer New York, New York, NY), pp. 165–188 (2014).
14. DC Koboldt, et al., VarScan: variant detection in massively parallel sequencing of individual and pooled samples. *Bioinformatics* **25**, 2283–2285 (2009).
